# A unified framework for estimating parameters of kinetic biological models

**DOI:** 10.1186/s12859-015-0500-9

**Published:** 2015-03-27

**Authors:** Syed Murtuza Baker, C Hart Poskar, Falk Schreiber, Björn H Junker

**Affiliations:** 1 Manchester Institute of Biotechnology, University of Manchester, Manchester, UK; 2 Systems Biology Group, Leibniz Institute of Plant Genetics and Crop Plant Research (IPK), Gatersleben, Germany; 3 Institute of Pharmacy, Martin Luther University, Halle, Germany; 4 Clayton School of Information Technology, Monash University, Clayton, VIC Australia; 5 Institute of Computer Science, Martin Luther University, Halle, Germany

**Keywords:** Constrained parameter estimation, Identifiability analysis, Kalman filter, Kinetic models, Parameter estimation framework

## Abstract

**Background:**

Utilizing kinetic models of biological systems commonly require computational approaches to estimate parameters, posing a variety of challenges due to their highly non-linear and dynamic nature, which is further complicated by the issue of non-identifiability. We propose a novel parameter estimation framework by combining approaches for solving identifiability with a recently introduced filtering technique that can uniquely estimate parameters where conventional methods fail. This framework first conducts a thorough analysis to identify and classify the non-identifiable parameters and provides a guideline for solving them. If no feasible solution can be found, the framework instead initializes the filtering technique with informed prior to yield a unique solution.

**Results:**

This framework has been applied to uniquely estimate parameter values for the sucrose accumulation model in sugarcane culm tissue and a gene regulatory network. In the first experiment the results show the progression of improvement in reliable and unique parameter estimation through the use of each tool to reduce and remove non-identifiability. The latter experiment illustrates the common situation where no further measurement data is available to solve the non-identifiability. These results show the successful application of the informed prior as well as the ease with which parallel data sources may be utilized without increasing the model complexity.

**Conclusion:**

The proposed unified framework is distinct from other approaches by providing a robust and complete solution which yields reliable and unique parameter estimation even in the face of non-identifiability.

**Electronic supplementary material:**

The online version of this article (doi:10.1186/s12859-015-0500-9) contains supplementary material, which is available to authorized users.

## Background

Systems biology integrates computational modelling with experimental techniques in order to better understand the function of living organisms, the regulation of their cellular processes and how these cells react to environmental perturbations [1]. Among the different computational approaches, kinetic modelling gives the most detailed representation of the biological system. These models build on the stoichiometry of the reactions, incorporating the dynamic interactions between different components of the network. The dynamics in kinetic models are driven through ordinary differential equations (ODEs) that represent the internal reaction mechanism as a function of species concentration and parameters. These model parameters play a crucial role in describing the correct dynamics of the model. However, it is only possible to measure a fraction of these kinetic parameters in wet lab experiments due to high cost, difficulty and limitations in current techniques or methods [2]. Therefore these parameters are indirectly determined through computational methods from other measurement quantities, in particular the time course data of metabolite concentrations. However, as biological models are often multi-modal it is not uncommon for traditional parameter estimation methods to become stuck in local optima [3]. In addition, traditional methods tend to perform badly in the presence of high measurement noise. Furthermore most of these methods do not consider any form of model uncertainty. Bayesian estimation is an alternative to traditional optimization techniques. This method considers both the system and measurement noise during the estimation. It calculates the posterior density of the parameter *θ* conditioned on observed data *y*. However, the calculation of this posterior involves high-dimensional integration for which no analytical solution is generally available. Therefore a numerical approximation has to be made for this posterior probability density. Among different Bayesian approaches, sequential methods have been shown to have a higher accuracy [4]. The widely used sequential Bayesian methods for parameter estimation are the sequential Monte Carlo (SMC), also known as particle filtering [5], and the Kalman filtering (KF) type methods. Particle filtering is computationally expensive due to the calculation of several hyper-parameters [6]. This makes it unsuitable for large biological systems. The Kalman filter has the capability of using noise-corrupted measurement data and other inaccuracies to estimate the parameter values in a recursive manner, even when none of the variables are directly measurable [7,8]. In terms of computational cost, KF type approaches are more moderate. The Kalman filter was originally derived as a state estimator used to estimate the hidden state variables (i.e. variables that are not directly measurable). Within the KF framework, the parameter estimation problem can be reformulated as a state estimation problem, where it considers the parameters as hidden variables and tries to estimate their values [9]. The KF operates by approximating the probability density function of the parameters and can cope efficiently with multi-modality, asymmetries and discontinuities [10]. This is a very powerful technique which can perform estimation even when the precise knowledge of the model is not available or the measurement data is noisy and incomplete [11]. However, the basic KF is limited to linear systems whereas most biological models are non-linear. Several non linear extensions of the Kalman filter have been successfully used for parameter estimation in biological systems, of which the two most widely used are the extended Kalman filter (EKF) and the unscented Kalman filter (UKF) [2,3,9,12]. Among these two non-linear extensions, UKF has the better estimation accuracy due to its approach of handling the non-linearity [13-15]. However, UKF suffers from numerical instability when the estimation covariance matrix is not positive definite. Moreover, there are no general methods for introducing constraints into the estimation process in UKF, which is crucial in biological modelling to ensure biologically meaningful parameter values [16]. The square-root variation of UKF (SR-UKF) proposed by Merwe and Wan, 2001 solves the numerical stability problem of the UKF but does not have the mechanism to introduce constraints into its estimation procedure. Recently these issues have been addressed with the development of the constrained square-root unscented Kalman filter (CSUKF), a constrained extension of the SR-UKF, which was specifically designed for use with biological models [17]. The CSUKF estimates the parameters within a biologically meaningful parameter space while guaranteeing numerical stability of the filtering technique by ensuring positive definiteness of the covariance matrix.

A second issue that arises in the successful parameter estimation for any kind of model is non-identifiability [18]. Identifiability analysis tries to answer the question of whether or not it is possible to have a unique estimation of an unknown parameter within the constraints of the mathematical model, the available measurement data and the corresponding level of error (noise) in this data [19]. For a non-identifiable model, different sets of parameter values agree equally well with the measurement data which results in an un-reliable model [20]. Such models might not address the underlying biological question properly, thus reducing any value derived from the model. Therefore it is reasonable to perform parameter estimation only after non-identifiability within the model has been determined and resolved. Non-identifiability can be divided into two types, structural and practical non-identifiability [21]. If the non-identifiability in the parameter arises due to the model structure then it is called structural non-identifiability, whereas if it is due to measurement data it is called practical non-identifiability. For successful parameter estimation it is necessary to address both types of non-identifiability.

In this paper we propose an integrated approach to form a novel parameter estimation framework, leveraging the inherent features of the CSUKF in combination with techniques in identifiability analysis. This approach combines two modules, the first for parameter estimation, centering on the CSUKF and the second for identifiability analysis (IA). The IA module encompasses a data-oriented identifiability analysis that categorizes both structurally and practically non-identifiable parameters. To assist in resolving any non-identifiability, the framework includes ranking of the parameters and the determination of the correlation and functional relationship(s) involving non-identifiable parameters. These features provide feedback that guide the design of both the model and experiment to solve the problem of non-identifiable parameters. However, under real world situations it is not always possible to solve the non-identifiability outright, which typically requires acquiring additional data or simplifying the model. Often the required additional measurement data is either not available or not technically possible. Furthermore model simplification may significantly limit the ability for generating predictive behavior, reducing the usefulness of the model. Thus for a complete solution the framework includes a novel method for estimating parameters even in the presence of non-identifiability. This method uses the informed prior to formulate the prior state distribution for the CSUKF which subsequently allows the CSUKF to determine a unique parameter estimation for a model which is otherwise non-identifiable from the frequentists perspective.

### Implementation

#### Model representation

Biochemical networks are nonlinear and dynamic in nature. In order to apply the CSUKF for parameter estimation of these biochemical networks, the system has to be formulated as a non-linear state space model [9]. In a state space model, the dynamics of the network are represented by a set of first-order differential equations in order to provide a powerful and convenient representation of the system. This representation consists of state variables and observed variables along with their different components and interactions. The total state of a system at any given time is represented by the state variables. The observed variables represent the values that are directly measurable in the system. Model quantities that are not directly observable are called hidden states. In this paper the following state space equation is used to represent the systems

1 $$ \begin{array}{l}\begin{array}{cc}\hfill \dot{x}=F\left(x,\theta, t\right)+w\hfill & \hfill, x\left({t}_0\right)\hfill \end{array}=x(0)\\ {}y=H\left(x,\theta, t\right)+v\end{array} $$

The vector *x* = [*x*_0_, *x*_1_, …, *x*_*n*_] represents the state of the system at any time *t* ≥ *t*_0_, with an initial value of *x*(0). The state vector is composed of the variables that are time dependent such as the concentration of proteins or metabolites. The state equation *F* defines the evolution of the state variables over time. In addition to the states, *F* is dependent on the model parameters, *θ* = [*θ*_0_, *θ*_1_, …, *θ*_*n*_]. The network may only be partially observable and so *x* may not be fully accessible. Thus the state variables can only be observed through the observation equation *H* where the output signals *y* is the quantity we can measure. The state equation is corrupted by process noise *w* which is an uncorrelated Gaussian white noise with probability distribution *p*(*w*) ~ *N*(0,*Q*). This noise describes the amount of confidence we have in our model. The measurement noise *v* with probability distribution *p*(*v*) ~ *N*(0, *R*) is also uncorrelated Gaussian white noise and similarly describes the reliability of the measurement data. Both the process noise covariance matrix *Q* and the measurement noise covariance matrix *R* are considered additive and positive definite.

### Parameter estimation in non-linear state space

The state-space definition can be extended to facilitate simultaneous state and parameter estimation by treating the parameters as augmented states *x* ^*aug*^ = [*x θ*] [12,22]. The dimension of this augmented state is the sum of the number of states and number of parameters. These parameters are constant values in the model with a 0 rate of change. Thus the parameter estimation problem becomes a state estimation problem, described by

2 $$ \begin{array}{l}\begin{array}{cc}\hfill \dot{x}=F\left(x,\theta, u,t\right)+w\hfill & \hfill, x\left({t}_0\right)=x(0)\hfill \\ {}\dot{\theta}=0\hfill & \hfill, \theta \left({t}_0\right)=\theta (0)\hfill \end{array}\\ {}y=H\left(x,\theta, t\right)+v\end{array} $$

### Deriving non-linear state space from ODEs

The dynamics of the biological systems are characterized by a set of ODEs. In order to represent the ODEs with state space equations they must first be cast into discrete form via the functions *f* (*k*), *k* ≥ 0 [23], which numerically integrates the state dynamics between the time points in which the state is observed.

3 $$ \begin{array}{rcl}f\left({x}^{aug}(k)\right)& =& {x}^{aug}(k)+{\displaystyle {\int}_{t_k}^{t_{k+1}}F\left({x}^{aug}\left(\tau \right)\right)d\tau}\\ {}{x}^{aug}\left(k+1\right)& =& F\left({x}^{aug}(k)\right)+w(k)\end{array} $$

where $$ {x}^{aug}(k)=\left[x(k)\begin{array}{cc}\hfill \hfill & \hfill \hfill \end{array}\theta \right] $$ is the augmented state vector at iteration *k*. For notational simplicity the discrete form of the augmented state vector *x* ^*aug*^(*k*) will be denoted *x*(*k*) throughout the remainder of this work.

Using this formulation the parameter estimation problem is restated as a state estimation problem, which can now be addressed within the framework of control theory using an extension to the Kalman filter.

### Overview of the framework

**Figure 1:**
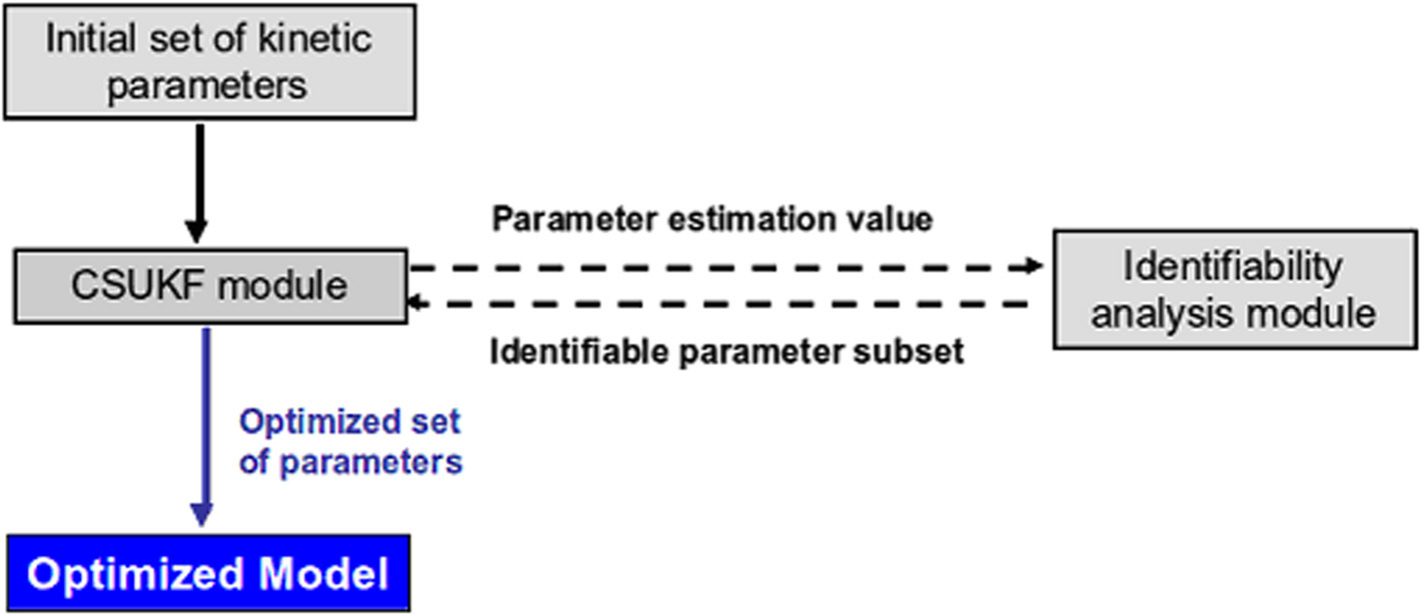
**Overview of the complete parameter estimation framework.** The estimation process begins by presenting the parameter estimation module with an initial set of kinetic parameters. A first pass through the parameter estimation is performed and used to initialize the identifiability analysis (IA) module. The IA determines and classifies the non-identifiable parameters, suggesting possible solutions. The IA then returns the subset of parameters found to be identifiable in addition to the informed prior which may be used by the CSUKF to formulate the prior state distribution. Thus the CSUKF is able to determine a unique solution, even if some parameters remain non-identifiable.

The main objective of this paper is to develop a complete parameter estimation framework around a novel filtering technique to successfully estimate parameters of biological kinetic models. The complete framework depicted in Figure 1 comprises two main modules, 1) the parameter estimation or CSUKF module and 2) the identifiability analysis (IA) module. Designed and implemented separately, the identifiability analysis nonetheless includes functions that are data driven, requiring a high degree of interaction with the parameter estimation module.

The IA is initially utilized to determine and classify non-identifiable parameters. Once found, the operation of the IA turns to resolving this problem of non-identifiability through a variety of operational sub-units. These sub-units perform a ranking of the parameters, and determine their correlation and functional relationships. The last step has the IA return the sub-set of parameters that may now be optimized for a unique solution, including the informed prior (if required) to work with any remaining non-identifiable parameters.

As the IA is data driven, the parameter estimation module is used to provide sets of partially optimized parameter values as initial values (in addition to other information such as the residuals. Once control is passed back to the estimation module, the CSUKF begins its basic operation of parameter estimation, starting with small random values. This estimation is iteratively refined until the predefined stop criterion is met, such as the number of iterations or the objective function reaching a stable or threshold value. Finally the optimized parameters are combined with the model yielding the optimized model.

In the next sections the two modules are described, starting with the parameter estimation module. The CSUKF will be briefly described, highlighting how it interacts with the identifiability analysis module. This is then followed by a detailed description of the identifiability analysis module.

### Parameter estimation module

Parameter estimation is performed using the constrained square-root unscented Kalman filter (CSUKF) [17]. Although it can stand on its own, this filtering technique was developed specifically to work within this greater framework. To this end it is numerically stable, can estimate parameters of a non-linear model and has the capability of introducing constraints into the estimation process. Its joint state and parameter estimation capability makes it possible to estimate parameters even in the presence of hidden variables. It takes into consideration both the process noise, due to model uncertainty, and measurement noise, due to error in the measurement data. The CSUKF applies the Bayesian framework to estimate the parameter values of biological models where reasoning under uncertainty is essential. While the introduction of constraints to this probabilistic inference technique results in more biologically meaningful parameter estimates.

### Parameter estimation with CSUKF

The CSUKF approximates the posterior probability of the state variable *x*(*k*), i.e. *p*(*x*(*k*)|*y*(*k*)), given the measurement data up to the time *k*. The posterior mean and covariance from this distribution are optimally calculated within the state constraint, *L*(*k*) ≤ *x*(*k*) ≤ *U*(*k*), where *L*(*k*) is the vector of lower bounds and *U*(*k*) is the vector of upper bounds. The UKF works by transforming the non-linear model to a statistically linear one and then applies the KF. This transformation is based on a minimal set of sample points, called sigma points, around the mean. The CSUKF guarantees these sigma points, and thus the mean, respect the boundary conditions by properly weighting them. These weights *W* ^*m*^ and *W* ^*c*^ are then adjusted according to the position of these sigma points. Numerical stability of the algorithm is ensured by propagating the square-root of the covariance matrix instead of the full covariance matrix.

These features make CSUKF a strong parameter estimation method for biological systems. For the complete algorithm and detailed explanation of the CSUKF see [17]. In addition to the general estimation, the CSUKF is used to generate parameter estimates for the methods in the IA module. This includes the initial parameter estimation for the data driven identifiability analysis and generating the trajectories for the profile likelihood based parameter identifiability analysis.

### Identifiability analysis module

Given a mathematical model and the associated measurement data, identifiability analysis determines whether it is possible to produce a unique solution for the unknown parameters [24]. Identifiability analysis is particularly significant for biological models as it determines the extent to which the same parameter value is reproducible in the face of noisy and limited measurement data [20,25]. Thus it is only reasonable to perform parameter estimation once identifiability issues have been resolved. To this end, the identifiability analysis module of the framework first determines the non-identifiable parameters of the model, classifies them and then directs the solution, either directly or indirectly (i.e. via the informed prior).

**Figure 2:**
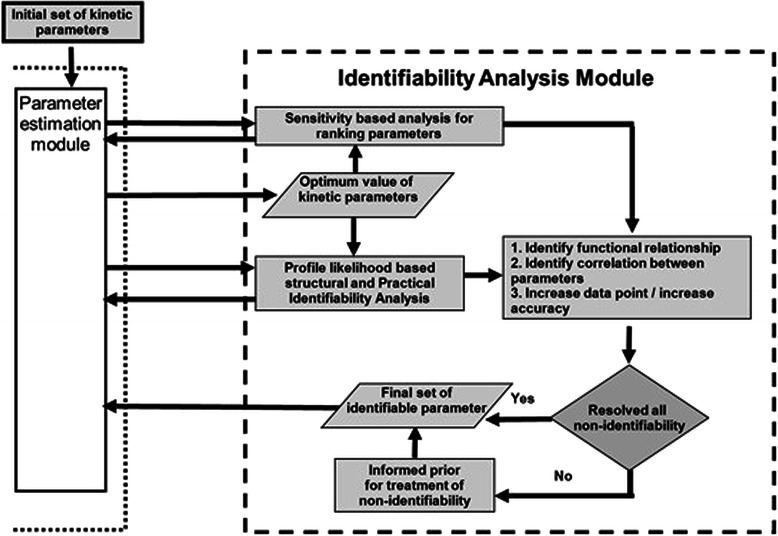
**The identifiability analysis module.** This module determines, classifies and solves (if found) non-identifiable parameters. The issue of non-identifiability is attacked two fold, directly via a ranking of the parameters and identification of both correlation(s) and non-linear functional relationship(s) between parameters, and indirectly via the generation of the informed prior. This detailed schema highlights the data driven nature of the identifiability analysis via the multiple interconnections between the IA and the parameter estimation module.

The identifiability analysis module is described in detail in Figure 2. The functionality of this module is divided into three main steps, analysis/classification, direct solution and indirect solution. The data driven identifiability analysis receives the initial set of parameter values together with residual values from the CSUKF in order to determine which, if any, parameters are non-identifiable. During the analysis, non-identifiable parameters are classified as being either structurally or practically non-identifiable. After finding the non-identifiable parameters, the IA module computes a sensitivity based ranking of the parameters. This ranking lists the parameters according to their importance. A common cause of non-identifiability is a linear or non-linear relationship between parameters. Linearly correlated parameters are identified through the correlation method and non-linear relationships among the parameters are ascertained by determining their functional relationship. Information on these specific relationships may then be used to determine possible solutions for non-identifiability among these parameters. In such relationships, parameters with high ranks are given priority for direct measurements in wet lab experiment. Using these new values, the low ranking parameters are re-evaluated to determine if they are still non-identifiable. When additional wet lab data is not available for any of the high or low rank parameters, the low ranking parameters may be set to small nominal values. This effect is minimal due to the lower sensitivity of these parameters on the system output [26]. The non-identifiability of the high ranking parameters is then re-evaluated, and if necessary the model may be reformulated to reduce the number of states and parameters as outlined in [27]. This type of simplification is targeted to solve the structural non-identifiability of the model. However this approach is only feasible if such simplification does not lead to a deletion of a pathway or reaction required for the targeted study of the model.

To solve the remaining practical non-identifiability the state trajectories are plotted along the parameter values to identify where the parameter uncertainty causes larger deviation in the state trajectory. This identifies where an increase in either the number of data points or the accuracy of the existing data would help to resolve the practical non-identifiability. However, it is often the case with biological systems that an increase in the quantity or accuracy of the measurements is not a practical solution.

For any remaining non-identifiable parameters the indirect solution is applied. The CSUKF is a Gaussian estimation procedure where the posterior probability distribution of a state variable is calculated from its prior distribution and the likelihood. This prior probability distribution expresses the subjective uncertainty about the state variables before utilizing the measurement data. An informed prior can be formulated if there is previous information regarding the distribution of the state variable in question [28]. The determination of an informed prior for a state variable allows the CSUKF to produce a unique estimation.

The following sections provide more detail on each of the specific functions comprising the identifiability analysis module shown in Figure 2.

### Parameter ranking calculation

When considering solutions to non-identifiable parameters, it is beneficial to first determine the sensitivity of individual parameters. Parameters having high sensitivity towards the state variables must be estimated accurately. However, parameters with sensitivity below a critical threshold essentially have little or no effect on the model. This framework utilizes the orthogonal based parameter ranking method [26,29]. This is a data driven method that calculates the ranking based on the estimated parameter values. The sensitivity matrix is formed by taking the partial derivative of the system state output with respect to each of the model parameters. Elements of this matrix, denoted as sensitivity coefficients, are then used to measure the effect of the change in a parameter on the system output. This orthogonal based method ranks the parameters based on their sensitivity and linear independence with respect to the other parameters. The sensitivity matrix, denoted Z^a^, is given by

4 $$ {Z}^a=\frac{\partial X}{\partial \Theta}=\left[\begin{array}{cccc}\hfill {z}_{1,1}^a\hfill & \hfill {z}_{1,2}^a\hfill & \hfill \cdots \hfill & \hfill {z}_{1,n}^a\hfill \\ {}\hfill {z}_{2,1}^a\hfill & \hfill {z}_{2,2}^a\hfill & \hfill \cdots \hfill & \hfill {z}_{2,m}^a\hfill \\ {}\hfill \vdots \hfill & \hfill \vdots \hfill & \hfill \ddots \hfill & \hfill \vdots \hfill \\ {}\hfill {z}_{n,1}^a\hfill & \hfill {z}_{n,2}^a\hfill & \hfill \cdots \hfill & \hfill {z}_{n,m}^a\hfill \end{array}\right] $$

where *X* is the vector with all output elements, Θ is the parameter vector and ${z}_{i,j}^a=\frac{\partial {x}_i}{\partial {\theta}_j}$ is the sensitivity of state *i* with respect to parameter *j*. In order to normalize the effect of high state or parameter values, individual elements of the matrix are scaled as

5 $$ {z}_{i,j}=\frac{\partial {x}_i}{\partial {\theta}_j}.\frac{{\widehat{\theta}}_j}{{\widehat{x}}_i} $$

where ${\widehat{\theta}}_j$ is the optimal estimate of the *j* ^*th*^ parameter and ${\widehat{x}}_i$ is the value of the *i* ^*th*^ output variable.

The parameters are then ranked using the orthogonal based algorithm described by [26], based on their sensitivity towards the model output. This ranking selects the parameter with the largest orthogonal distance from the rest of the parameters in their sensitivity matrix as having the highest impact on the model response with the maximum linear independence. The net influence of the selected parameter on each of the remaining parameters is adjusted by regressing the original columns of the sensitivity matrix on to the column associated with the selected parameter. The next parameter is chosen based on a residual value calculated from the orthogonal distance between the sensitivity matrix and the regression matrix. The algorithm is presented in detail in Additional file 1.

In this framework the ranking information is used in combination with the other tools in the IA module to better target solutions. However in some applications the ranking is used as a direct indication of identifiability based on a predetermined threshold. As demonstrated, in the analysis of the sugar cane culm model, while the ranking provides useful information, it is unreliable as the sole indicator of identifiability.

### Profile likelihood based structural and practical identifiability analysis

In the Kalman filter, and its non-linear variants, parameter identifiability is typically addressed in the view of observability [12]. However, since the computational complexity of this analysis increases with both non-linearity and model size, this analysis is not well suitable for large scale biological models. In order to better target biological modelling, our framework integrates the profile likelihood based identifiability analysis [21] to determine both the structural and practical non-identifiable parameters. In parameter estimation a weighted sum of squared residual (the difference between estimated and measured data) is commonly minimized to estimate the parameter values. For normally distributed measurement noise, this difference follows a *χ* ^2^ distribution when evaluated at the optimal solution [30] and corresponds to the maximum likelihood estimation of the parameters [20]. A robust confidence region is then derived from the asymptotic *χ* ^2^ distribution of the likelihood ratio test by calculating the profile likelihood of the parameters [31,32]. To use the confidence interval, the profile likelihood trajectory is calculated for each parameter *θ*_*i*_ along the minimum of the *χ* ^2^ (*θ*) with respect to all other parameters. Then for each parameter, the corresponding trajectory is compared to the *θ*_*j*≠*i*_ desired confidence interval, a threshold of 95% (i.e., approx. 2 standard deviations), to determine if the parameter is structurally or practically non-identifiable.

Essentially the profile likelihood method explores the space around each parameter in the direction of least increase of *χ* ^2^ (*θ*). This method reduces the maximum likelihood estimation to a function of a single parameter of interest by considering the other parameters to be nuisance parameters. Nuisance parameters are those parameters which are not of direct interest but are required for the successful analysis of the parameter of interest. In its calculation the parameter vector is partitioned as *θ* = (*ψ,η*) where *ψ* is the vector of parameters of interest and *η* is the vector of nuisance parameters. The parameter of interest is kept fixed at its optimal value and the nuisance parameters are varied to produce the maximum likelihood (ML) trajectory. The profile likelihood at step *k* is defined as

6 $$ p{l}_k=\underset{\eta }{ \max }{l}_k\left(\psi, \eta \right) $$

where *l*_*k*_(*ψ,η*) is the maximum likelihood estimation of the parameter *ψ* maximized over *η* at the *k* ^th^ step of the profile likelihood calculation.

The profile likelihood trajectory can be used to build a confidence region for each of the parameters individually. This confidence interval is called the likelihood based confidence region which is based on the generalized likelihood ratio test [31]. This likelihood ratio test follows an asymptotic *χ* ^2^ distribution. Considering $l\left(\widehat{\theta}\right)$ as the maximum likelihood estimation (MLE) and *pl*(*θ*) as the profile likelihood of the parameter vector *θ*, then the likelihood ratio is written as

$$ 2\left[ pl\left(\theta \right)-l\left(\widehat{\theta}\right)\right]<{\Delta}_{\left(\alpha, m\right)} $$

where Δ_(*α*,*m*)_ is the threshold value for 1-α quantile of *χ* ^2^ distribution with *m* degrees of freedom. Following a *χ* ^2^ distribution, the equation can be rewritten as [19]

7 $$ \left({\chi}^2\left(\theta \right)-{\chi}^2\left(\widehat{\theta}\right)\right)<{\Delta}_{\left(\alpha, m\right)} $$

where *χ* ^2^(*θ*) represents the objective function value of the profile likelihood and ${\chi}^2\left(\widehat{\theta}\right)$ is the MLE of the parameter vector, both calculated while keeping the parameter of interest fixed to a predefined value. The border of this confidence region represents the likelihood confidence interval [21]. To calculate this profile likelihood trajectory we start with the initial optimal solution of the parameter values calculated using the CSUKF. In combination, the KF together with this identifiability analysis has a likelihood interpretation with equations derived from the chi-square merit function [33]. Using the representation of *χ* ^2^ in vector form and the notations from the CSUKF derivation, the same *χ* ^2^ merit function used for the sum of squared residual can be used for the CSUKF at the k^th^ iteration as

$$ {\chi}_k^2=\left(y(k)-{\widehat{y}}^{-}(k)\right){R}^{-}{\left(y(k)-{\widehat{y}}^{-}(k)\right)}^T $$

Thus the final merit function is

8 $$ {\chi}^2={\displaystyle \sum_{k=1}^n\left(y(k)-{\widehat{y}}^{-}(k)\right)}{R}^{-}{\left(y(k)-{\widehat{y}}^{-}(k)\right)}^T $$

Where *n* is the number of data points, *R* is the observation error covariance matrix, *y*(*k*) is the vector of observation data and *ŷ* ^−^(*k*) is the current estimate of the observed state variables. The parameter for which we seek to calculate the profile likelihood is then increased step by step. The nuisance parameters are then optimized using the CSUKF to reach the global optima with the specific value of the fixed parameter. This parameter is increased until either the *χ* ^2^ crosses the threshold value (corresponding to a 95% confidence interval) or it is determined to run horizontal, i.e., not crossing the threshold. This represents the upper bound of the confidence interval. The same approach is applied again with decreasing step size starting at the optimal solution to calculate the lower bound of the confidence interval. This process is repeated for each parameter deriving each of their likelihood based confidence intervals. Based on the analysis they are defined to be identifiable, structurally non-identifiable or practically non-identifiable.

**Figure 3:**
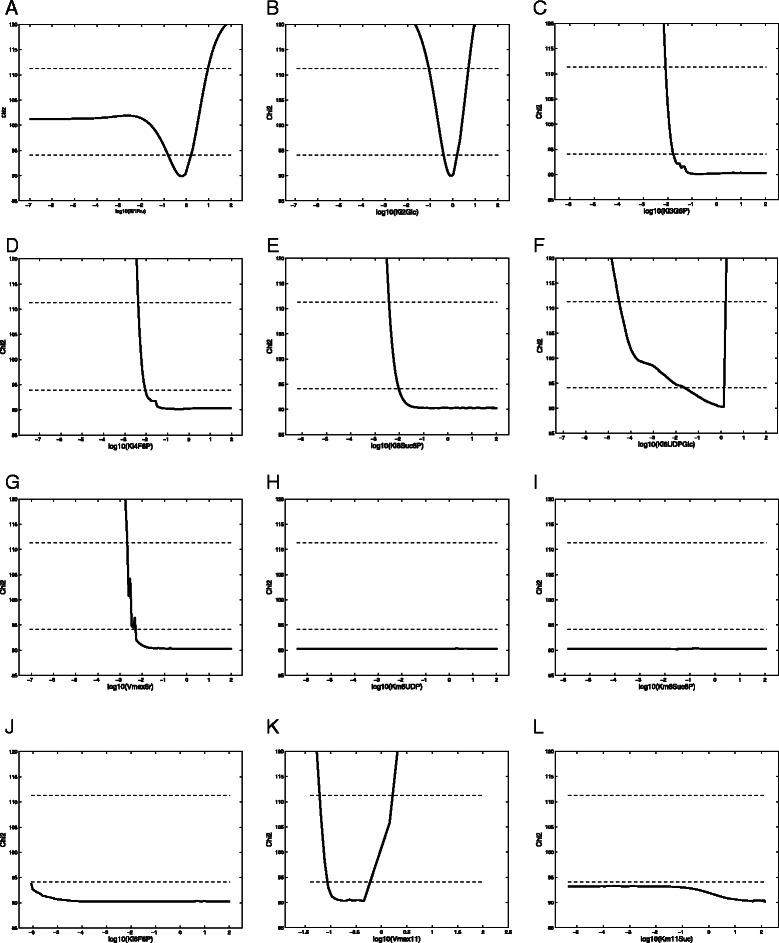
**Profile likelihood based parameter identifiability analysis for each of the 12 estimated parameters.** The solid line represents the profile likelihood trajectory, with the parameter values in log scale. Panels **(a) - (l)** represent the 12 parameters for which the identifiability analysis was conducted. In each plot the dotted lines represent the two thresholds. The lower threshold is the 95% point wise confidence interval and the upper threshold is the 95% simultaneous confidence interval. If the profile likelihood of a parameter crosses the threshold line for both high and low values then the parameter is identifiable. A horizontal (i.e., flat) profile likelihood indicates structural non-identifiability, while crossing the threshold(s) on only one side indicates practical non-identifiability. In most of the cases it is reasonable to conclude that the parameters crossing the pointwise confidence intervals are identifiable.

The *i* ^th^ parameter *θ*_*i*_ is said to be identifiable, if it has a finite likelihood based confidence interval, that is ${\sigma}_i^{-}>-\infty$ and ${\sigma}_i^{+}<+\infty$, where $\left[{\sigma}_i^{-},{\sigma}_i^{+}\right]$ are respectively the lower and upper bounds of the confidence interval. Conversely, when either one or both of the limits approach infinity, i.e., *χ* ^2^(*θ*_*i*_) does not cross the given threshold; the corresponding parameter cannot be estimated [20]. When a parameter has infinite confidence interval in both directions it is classified as structurally non-identifiable. However, if the confidence interval is infinite in only one direction, then it is classified as practically non-identifiable (see Figure 3 for examples).

Either type of non-identifiability may be solved by direct measurement of the parameters, However this is typically not a feasible solution, thus each type of non-identifiability may be attacked indirectly. Structural non-identifiability is due to an insufficient mapping of the observation function resulting from functionally related parameters [20]. As such structural non-identifiability is independent of the measurement data. Possible solutions are to alter the observation function by measuring different state variables [21] or to modify the model definition through simplification. On the other hand, practical non-identifiability depends on the amount and/or the accuracy of the measurement data. Therefore practical non-identifiability may be solved by an increase in the amount and/or the accuracy of the measurement data.

### Determining inter-related parameters

When there exists a relationship between two or more parameters, these parameters are non-identifiable [34]. However, if these relationships, classified as linear or non-linear, can be determined, the non-identifiability may be resolved for all affected parameters.

Linear relationships can be identified by analyzing the correlation between parameters. The conventional method uses the covariance matrix to calculate this correlation. The inverse of the fisher information matrix (FIM) is used to provide an estimation of the lower bound of the covariance matrix according to the Cramèr − Rao inequality [35]. However, when dealing with non-linear models the FIM may lead to a poor approximation [36]. In this framework, the correlation coefficient is calculated from the square-root of the state covariance matrix generated by the CSUKF during the parameter estimation process. The covariance matrix calculated by the sigma point method is highly accurate and does not require the calculation of gradients or the Jacobian [36].

Non-linear relationships cause the parameters to be functionally related. This framework incorporates the mean optimal transformation approach (MOTA) developed by [34] to uncover functionally related parameters. MOTA is a non-parametric bootstrap type algorithm, based on an optimal transformation of the dependent (response) variable and a set of independent (predictor) variables. This transformation is estimated by the alternating conditional expectation (ACE) [37], a non-parametric regression method used to explore the effect of one or more independent variables on the dependent variable.

### Informed prior for treatment of non-identifiability

The previous techniques of the identifiability module deal with determining non-identifiable parameters and suggesting solutions, such as which additional measurement data would help solve the non-identifiability. However situations frequently arise in systems biology where it is not possible to collect the required measurement data and simplification of the model may be undesirable or counter productive. In these scenarios the frequentists approaches, such as least squares, are incapable of estimation in the presence of non-identifiable parameters [28,38,39]. Thus, in the absence of identifiability these approaches cannot generate a unique set of estimated parameters. In contrast, Bayesian inference can make unique parameter estimation even in the presence of non-identifiability, provided that an informed prior distribution is provided [28,39].

Before discussing the informed prior, it is necessary to describe parameter identifiability from the perspective of a probability distribution. Given a set of parameters Θ and a vector of observed random variables *X* the conditional probability distribution of *X* given Θ is defined as *p*(*X*|Θ). If there exists two sets of parameters Θ_1_ ≠ Θ_2_ they are said to be non-identifiable if

9 $$ p\left(X\Big|{\Theta}_1\right)=p\left(X\Big|{\Theta}_2\right) $$

In other words, if the parameters are identifiable then two different sets of parameter values can not produce the same probability distribution [39].

However, an informed prior can be used to form a Bayesian inference for the parameters even if they are non-identifiable. As an example, let us consider a parameter vector with two elements, Θ = [*θ*_1_, *θ*_2_]. Different parameter values for the two sets of Θ are considered, where ${\Theta}^a=\left[{\theta}_1^a,{\theta}_2^a\right]$ and ${\Theta}^b=\left[{\theta}_1^b,{\theta}_2^b\right]$. The parameters can be uniquely identified with the use of an informed prior, e.g., *θ*_1 =_ *y* with probability 1 then Θ_1_ = Θ_2_ only when ${\theta}_2^a={\theta}_2^b$ making the model identifiable. Thus, if an informed prior is available, Bayesian inference is possible even for models which are otherwise non-identifiable from the perspective of likelihood. However by itself it is not sufficient to trust the solution from Bayesian inference. Without due care, such as an improper network definition or ill defined probabilities, Bayesian inference may not converge to the true value of a parameter [28]. As the CSUKF is an extension of dynamic Bayesian inference, the same approach can be applied to CSUKF. In CSUKF this proper prior is formulated by informedly initializing the state covariance matrix and the state noise covariance matrix.

## Results

To verify the applicability and accuracy of the proposed framework, it was implemented in the numerical tool-kit MATLAB and used to estimate parameters of two in-silico models, a kinetic model for sucrose accumulation in the sugar cane culm tissue [40,41] (SBML model available from the Biomodels database [42]), and a gene regulatory network supplied by the DREAM6 Estimation of Model Parameters Challenge [43] (the SBML model is available from the Sage Bionetworks’ Synapse database [44]^a^). Utilizing the Systems Biology toolkit, the models were converted from SBML to MATLAB as a system of ODEs. The framework was evaluated using synthetic measurement data generated by first simulating each model using all of the known parameters and then adding random Gaussian white noise to this simulated data. Despite starting with data generated directly from the known parameters, the information is lost between the movement of the parameter values to simulate the synthetic data and the return to parameters via estimation [45]. Thus the use of synthetic measurement data has become a general method to validate numerical algorithms [45].

### Experiment 1: The sucrose accumulation model in the sugar cane culm tissue

**Figure 4:**
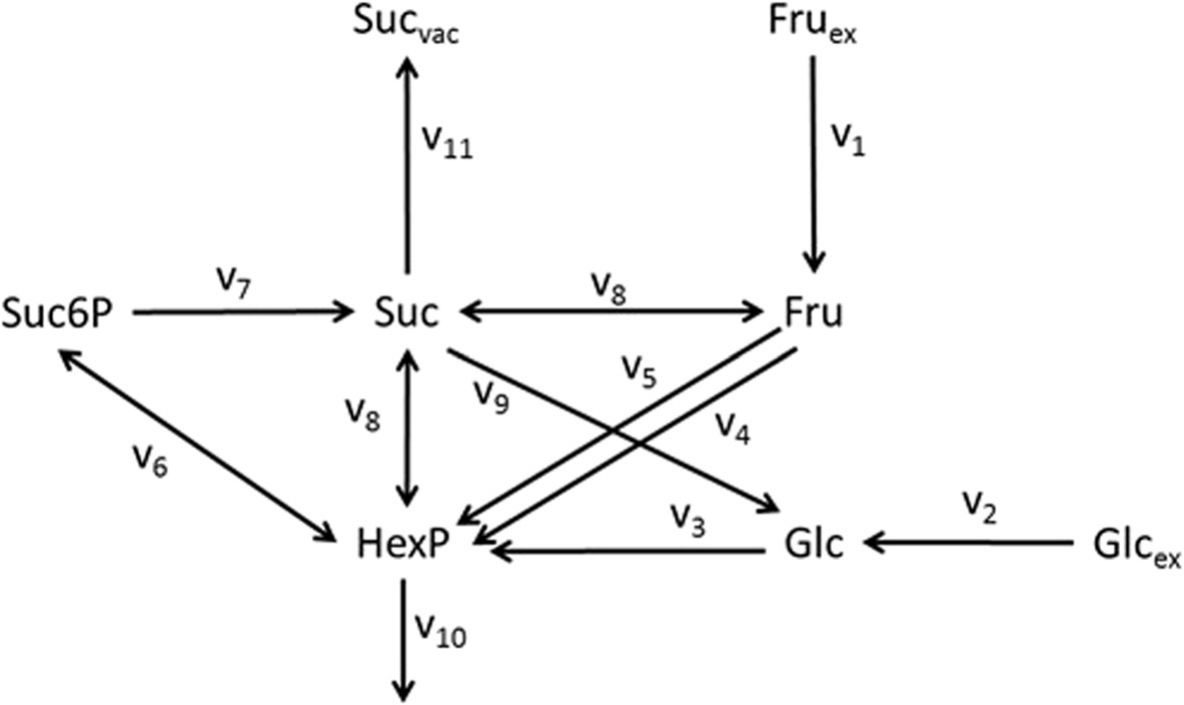
**Schematic diagram of the sucrose accumulation model of sugar cane culm tissue.** Abbreviations are as follows Suc: sucrose; Suc6P: sucrose-6-phosphate; HexP (Hexose phosphates); Fru: fructose; Glc: glucose. The subscript ‘ex’ stands for extracellular and the subscript ‘vac’ stands for vacuolar. The numbered V’s denote the reactions which are represented by rate laws. The reactions are V_1_: Fructose (Fru) uptake; V_2_: Glucose (Glc) uptake; V_3_: Hexokinase (Glc); V_4_: Hexokinase (Fru phosphorylating); V_5_: Fructokinase; V_6_: Sucrose phosphate synthase; V_7_: Sucrose phosphate phosphatase; V_8_: Sucrose synthase; V_9_: Invertase; V_10_: Glycolysis; V_11_: Vacuolar sucrose import.

Rohwer and Botha [40] published the kinetic model for sucrose accumulation in the sugar cane culm tissue which was then extended by [41] to account for isoforms of sucrose synthesis and fructokinase. The model helps to assess the biochemical control of sucrose accumulation and futile cycling in sugarcane. It provides the possibility of using different strategies to enhance sucrose accumulation and then selects the most promising one. The schematic diagram of the model is given in Figure 4. Details of the rate laws can be found in Additional file 1.

### Experimental setup

The model has 54 parameters from which 12 are selected for estimation, corresponding to the same 12 parameters that Rohwer estimated in his work [40]. The remaining 42 parameters are considered to be known and kept fixed throughout the estimation. Five metabolites have variable concentrations; Fru, Glc, HexP, Suc6P and Suc, while the rest are held constant. All five of these metabolites have an initial concentration of 1 mM. Synthetic time series data was generated for use as the measurement data, over the time interval [0 2340] seconds with a step size of Δ*t* = 10 seconds. The noisy measurement data was generated from the simulated time-series data *y*, as $$ {y}_{noisy}= \max \left[\begin{array}{cc}\hfill 0,\hfill & \hfill y\times \left(1+0.2\times r\right)\hfill \end{array}\right] $$, where *r* is a random variable having normal distribution with 0 mean and 1 standard deviation. The process noise covariance matrix *Q* is initialized with the augmented noise of the parameters and the state variables. The measurement noise covariance matrix *R* is initialized to 0.2 × *r* × *y*. The CSUKF is used to generate an initial approximation of the parameters as well as the datasets used to conduct the ranking and identifiability analysis.

### Orthogonal identifiability analysis and ranking

In this paper an orthogonal based ranking method is used to rank the parameters based on their probability of being identifiable [46]. Table 1 summarizes the results with the estimation from 50 runs of CSUKF along with the ranking of the parameters chosen from the most common ranking of those 50 runs. The threshold of the stop criteria for the ranking method is 0.004. Seven out of 12 parameters in the estimation have a standard deviation greater than 100% of their mean values. Furthermore, the mean value of six of these parameters is greater than 1 standard deviation from the actual parameter value. Parameters with high sensitivity (i.e., higher ranked parameters) must be well estimated as by definition the system is most sensitive to small variations in these parameters. For example, V_max6r_ which is ranked first) has the highest magnitude in the sensitivity coefficient matrix and thus the system is most sensitive to any variation in this parameter. On the other hand variations within low ranking parameters have substantially less effect on the system. Thus the high deviation of the estimate of parameter K_m6Suc6P_ (rank 2) is of more concern than the similar deviation of K_m6UDP_ (rank 6).

**Table 1:** **Parameter estimation results using the CSUKF, parameter ranking and profile likelihood analysis from the sugarcane model**

		**CSUKF**			
**Parameter name**	**Actual value**	**Mean**	**Std. Dev.**	**Orthogonal ranking**	**Proflile likelhood analysis**
V_max6r_	0.2	0.34	0.670	1	Practically NI
K_m6Suc6P_	0.1	5.97	4.580	2	Structurally NI
K_i6UDPGlc_	1.4	0.32	0.400	3	Identifiable
K_i1Fru_	1	1.00	0.010	4	Identifiable
K_i3G6P_	0.1	0.67	1.460	5	Practically NI
K_m6UDP_	0.3	4.73	3.450	6	Structurally NI
V_max11_	1	0.28	0.190	7	Identifiable
K_i6Suc6P_	0.07	0.45	0.770	8	Practically NI
K_i2Glc_	1	1.00	0.009	9	Identifiable
K_i4F6P_	10	0.63	0.850	N.I.	Practically NI
K_i6F6P_	0.4	0.65	1.060	N.I.	Practically NI
K_m11Suc_	100	21.43	21.820	N.I.	Practically NI

N.I. - Not Identifiable.

The mean and standard deviation of the estimated parameters are calculated from 50 repetitions. The ranking is chosen based on the weighted average ranking from each of the 50 runs. The profile likelihood analysis determines all non-identifiable parameters and classifies the non-identifiability as practical or structural. In each repetition the parameters are randomly initialized to values between 0 and 1.

As we will see, the relatively poor estimation, is due to several of the parameters being non-identifiable, which affects the estimation of all of the parameters. This allows the values of the parameters to vary within a wide range. Furthermore these parameters may affect the estimation of other parameters when the non-identifiability is due to a functional relationship between the parameters. This is more fully discussed in Additional file 1 with an example of functional relationships.

### Profile likelihood based analysis

The orthogonal identifiability analysis has several drawbacks, chief among them that it cannot conduct a full identifiability analysis. One indication of this is the relatively high standard deviations of the high ranking identifiable parameters, specifically the two parameters V_max6r_ (nearly 200% of the mean value) and K_m6Suc6P_ (77% of the mean value) in Table 1. One point to note is that this analysis depends on the initial value of the parameters. In some cases these parameters have high initial values at the beginning of the estimation which then decreases with the number of iterations [26]. Thus sensitivity analysis alone is not sufficient to perform a full identifiability analysis of a system. To this end, a profile likelihood based identifiability analysis is used to identify both the structural and practical non-identifiable parameters, by calculating the profile likelihood trajectories using data from the CSUKF. For this sugarcane model with 12 parameters and 234 data points, a good data agreement is found with an objective function value of *χ* ^2^ = 90.27. The step size is adjusted based on both the parameters and their profile likelihood values. When the profile likelihood trajectory is not smooth, a smaller step size is chosen. The step size is increased if the iteration stops prematurely, e.g. due to reaching the maximum number of iterations. For these 12 parameters the result of the profile likelihood identifiability analysis using a confidence interval of 95% is depicted in Figure 3. Defining the point-wise confidence interval threshold (i.e. when the degree of freedom is one) for a 95% confidence level is Δ_(*α,m*)_ = 3.84 and the simultaneous confidence interval threshold (i.e., when the degree of freedom is equal to the number of parameters) is Δ_(*α,m*)_ = 21.03.

As shown in Figure 3, only four of the parameters are actually identifiable, K_i1Fru_, K_i2Glc_, K_i6UDPGlc_ and V_max11_, with finite likelihood based confidence intervals in both the increasing and decreasing directions of the parameter values. Two parameters are structurally non-identifiable, the more severe of the two, with completely flat profile likelihoods, K_m6Suc6P_ and K_m6UDP_. The elevated standard deviations, a feature associated with structurally non-identifiable parameter estimates [34], are, if anything misleadingly optimistic. In fact, structurally non-identifiable parameters can take any value within a wide range without having any affect on the objective function (recall the flat profile likelihoods’), and typically cannot be solved solely through additional measurements. Such non-identifiability is often due to the over-parameterization of the model [18], which may be due to functional relationships among the parameters of the model [39].

The remaining parameters, K_i3G6P_, K_i4F6P_, K_i6Suc6P_, V_max6r_, K_i6F6P_ and K_m11Suc_, were found to be practically non-identifiable with their likelihood-based confidence region extending infinitely in one direction (Figure 3). This indicates that these parameters cannot be reliably estimated with acceptable accuracy from the available noisy measurement data [20,21,47].

### Solving parameter non-identifiability, parameter reduction and targeted measurements

After the appropriate categorization of all parameters, these non-identifiabilities must be solved to have a unique parameter set. The simplest approach to solve the structural non-identifiability of the parameters is to directly measure them. To minimize or eliminate parameter measurements there are methods which try to change the model structure in order to remove over parameterization. This includes changing the mapping of the observation function through new measurement data [19,21] or to use a known functional relationship. In the latter case only a subset of the functionally related parameters need to be directly solved. In this case the higher ranked parameters are measured while the lower ranked parameter(s) remain estimated or when parameter measurements are not possible, the high ranking parameters are estimated while keeping the low rank parameter(s) fixed to a nominal value [26].

In this framework, we first try to determine whether the two structurally non-identifiable parameters have a linear or non-linear relationship with any other parameter(s), then take guided action. The mean optimal transformation approach (MOTA) using the profile likelihood estimation data of the two structurally non-identifiable parameters was applied to determine any functional relationships. MOTA identified functional relationships for both of these parameters, K_m6UDP_ and K_m6Suc6P_. Parameter K_m6UDP_ was found to have two functional relationships, one with K_i3G6P_ and one with V_max6r_. The second structurally non-identifiable parameter, K_m6Suc6P_ was also found to be functionally related to V_max6r_. Since V_max6r_, which was determined to be practically non-identifiable, is also the highest ranking parameter, it is targeted for measurement. Thus in this example the measurement of a single parameter, V_max6r_, solves the structural non-identifiability of both K_m6UDP_ and K_m6Suc6P_. A more detailed discussion on function relationship is given in Additional file 1.

**Figure 5:**
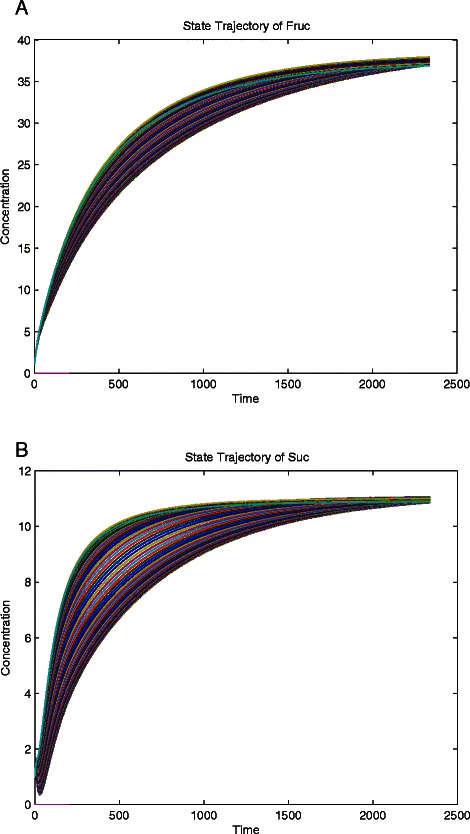
**Solving K**_**m11Suc**_ **.** The two plots are trajectories of concentration vs. time for **a)** Fru and **b)** Suc plotted over the range of the values of K_m11Suc_ generated during the calculation of the profile likelihood. Places of larger variability denote points where new measurement would efficiently estimate the parameter.

Practical non-identifiability is typically due to an insufficient amount and/or quality of measurement data, [19,21]. The model trajectories of the state variables along the profile likelihood of the practically non-identifiable parameters are examined to determine which measurements are needed to solve the practical non-identifiability. An example of these trajectories is illustrated in Figure 5. This is used to identify the points where the uncertainty in a specific parameter has the largest impact on the model uncertainty. Thus regions of high variation within these trajectories help to identify which measurements will have the largest impact on the model uncertainty [20]. A second cause of practical non-identifiability is correlation between parameters [48,49]. The flattening of the trajectory of a practically non-identifiable parameter may be due to the correlation with one or more other parameters. The non-identifiability among two or more correlated parameters requires measurement data for all but one of the correlated parameters to be available. Guedj et al. [50] discussed a similar approach where they analyzed the practical identifiability of a dynamic model of HIV through the correlation of the parameters. At each iteration the CSUKF estimates both the mean and the square-root of the covariance. From this the correlation coefficient matrix is calculated, and used to guide the targeting of parameters to be measured.

The analysis found a strong correlation between K_i3G6P_ and K_i4F6P_. It is not possible to use the ranking to select between K_i3G6P_ and K_i4F6P_ as the latter was found to be non-identifiable during the orthogonal ranking. However, as both techniques identified parameter K_i4F6P_ as non-identifiable, it was selected for measurement. A significant correlation was also found between K_i6F6P_, V_max6r_ and K_i6UDPGlc_. Among these three parameters K_i6UDPGlc_ is an identifiable parameter and V_max6r_ has already been picked up for measurement. In the best case this would also solve the non-identifiability of K_i6F6P_, however this parameter remained non-identifiable and therefore was additionally selected for measurement.

Of the remaining two unidentifiable parameters, K_m11Suc_ and K_i6Suc6P_, the state trajectories of each concentration were plotted over the range of profile likelihood values of these parameters. This analysis revealed variations in the states of fructose and sucrose, Figure 5(a) and (b) respectively, over the profile likelihood values of K_m11Suc_. This trajectory suggests a large variation in state trajectories, for both uptakes, which indicates that new measurement data for these states may solve the practical non-identifiability of K_m11Suc_. Thus new synthetic measurement data was generated with a smaller time step of 0.25 seconds.

**Figure 6:**
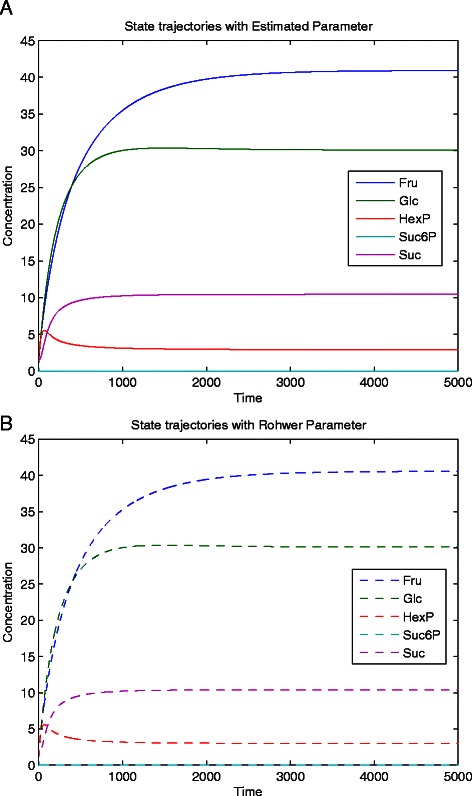
**Simulation of the state dynamics in the Sugarcane calm model. a)** Simulation based on the values 9 estimated and 3 measured parameters. **b)** Simulation based on the actual parameter values.

The analyses did not find any explicit relationships for the last non-identifiable parameter, K_i6Suc6P_. However, it was found that the preceding measurements were sufficient to solve this non-identifiability. It is thought that an as yet undetermined, more complicated, functional relationship exists among K_i6Suc6P_ and multiple other parameters. The results from utilizing these additional measurements are summarized in Table 2. By properly identifying and solving the non-identifiability through additional targeted measurements the estimated values more closely approach the original values. Furthermore it clearly illustrates that the CSUKF can accurately estimate the parameters once the issue of non-identifiability has been dealt with. The dynamics of the sugarcane model states were simulated using the newly estimated parameter values, see Figure 6. As expected, accurately estimated parameter values are able to reproduce not only a reasonable prediction of the stationary state, but are also able to accurately reproduce the dynamics of the system. However, solving the non-identifiabilities in the first place required additional measurement data for the metabolites or directly measuring the parameters. The next section illustrates the alternative when additional information is simply not available or even not possible.

**Table 2:** **Final parameter estimation result with confidence intervals after solving the non-identifiability**

**Parameter name**	**Original value**	**Value**	**σ+**	**σ-**
K_i1Fru_	1.00	0.99	1.19	0.18
K_i2Glc_	1.00	1.00	2.07	0.40
K_i3G6P_	0.10	0.10	0.11	0.10
K_i6Suc6P_	0.07	0.05	0.09	0.01
K_i6UDPGlc_	1.40	1.16	2.32	0.05
K_m6UDP_	0.30	0.40	0.63	0.18
K_m6Suc6P_	0.10	0.16	0.56	0.06
V_max11_	1.00	0.99	1.45	0.09
K_m11Suc_	100.00	99.59	102.48	96.70
*K_i4F6P_	10.00	10.00	-	-
*V_max6r_	0.20	0.20	-	-
*K_i6F6P_	0.40	0.40	-	-

*Parameters that were measured.

To achieve this, three non-identifiable parameters (K_i4F6P_, V_max6r_ and K_i6F6P_) were “explicitly” measured and the rest were estimated. During each successive estimation phase, parameters estimated with high confidence in a previous run are fixed. The asymmetric confidence interval has upper bound σ + and lower bound σ-.

### Results using the informed prior

While the typical course to solving non-identifiability is through additional measurements, the simple fact is that this is not generally feasible through biological experiments [51]. While the situation is continuously improving, such as recent developments in devices and protocols for measuring time series data, these datasets remain noisy and incomplete due to the ever increasing model complexity coupled with limitations in measurement techniques [52]. Thus it is not always possible to directly measure parameter values or to measure extra data points in the time-series data.

In such cases an accurate estimation requires alternative methods for solving non-identifiability. As the CSUKF is an extension of the Kalman filter it benefits from the ability to make use of an informed prior. Thus as an alternative to additional measurements this framework applies the informed prior treatment of the Bayesian approach to solve any remaining non-identifiability. In this approach an informed prior distribution is defined for the parameters in the IA module. This informed prior is provided to the CSUKF which utilizes it to uniquely estimate the parameters even in the case of non-identifiability. The CSUKF belongs to the Gaussian family, thus the conjugate prior distribution can be used to define the prior for the parameters and state variables, while maintaining the same probability density function (pdf) after transformation [53]. Lindley & El-Sayyad [54] applied a similar treatment for non-identifiable parameters, using Bayesian inference to estimate parameters with respect to linear constraints.

This approach was applied to the sugarcane model using the original synthetic measurement data and with the expectation that no extra experimental data can be measured to otherwise solve the non-identifiability. Thus not only must all twelve unknown parameters be estimated, but no additional time series measurement data is available for use.

During the estimation the informed prior is introduced into the distribution through the uncertainty of the parameter values. The square-root of the covariance matrix for the state estimation matrix *V* and the state noise covariance matrix *Q* are initialized with subjective uncertainty to formulate the prior. Initially the orthogonal based method finds the rank of the parameters. During the rank calculation the uninformed prior is used. Results from this ranking are then used to formulate the informed prior. Both *V* and *Q* are realized on the basis of the rank of the parameters, where high ranking parameters are more sensitive towards the model states and consequently are initialized with low standard deviations. Similarly the insensitive low ranking parameters are initialized with high standard deviations.

The results from the parameter estimation using the informed prior are summarized in Table 3, with statistics from 50 repetitions. Using the informed prior the resulting estimates are shown to have low standard deviations, with only two parameters having a deviation above 2% of its estimated mean value, K_i4F6P_ with 18.5% and K_i3G6P_ with 5%. Overall there is a decrease in the relative standard deviations of from one to three orders of magnitude. From this it is clear that by utilizing the informed prior this framework can uniquely estimate parameters even in the presence of non-identifiability. While this does not guarantee a corresponding improvement in estimation accuracy, all but two of the parameters show improvement in their estimation over the previous results without using the informed prior. What must be emphasized is that no additional data has been added, thus the parameter estimation yields a unique set of parameters that can best recreate the state space of the time series data and not the specific underlying values of the biological parameters. A steady state analysis with the estimated values using the informed prior verifies the in vivo behavior of the model by reproducing the distribution of metabolite concentrations (Table 4). This indicates that although some of the parameters were not close to the value described by Rohwer, these parameters are in good agreement for capturing the actual dynamics of the original system.

**Table 3:** **Results of parameter estimation using CSUKF with and without the informed prior**

			**CSUKF without informed prior**		**CSUKF with informed prior**	
**Parameter name**	**Original value**		**Mean**	**Std. Dev.**	**Mean**	**Std. Dev.**
K_i1Fru_	1.00		1.00	0.010	1.00	0.0100
K_i2Glc_	1.00		1.00	0.009	1.00	0.0100
K_i3G6P_	0.10	NI	0.67	1.460	0.16	0.0080
K_i4F6P_	10.00	NI	0.63	0.850	6.26	1.1600
K_i6Suc6P_	0.07	NI	0.45	0.770	0.25	0.0010
K_i6UDPGlc_	1.40		0.32	0.400	0.14	0.0005
V_max6r_	0.20	NI	0.34	0.670	0.07	0.0003
K_m6UDP_	0.30	NI	4.73	3.450	4.69	0.0550
K_m6Suc6P_	0.10	NI	5.97	4.580	3.49	0.0100
K_i6F6P_	0.40	NI	0.65	1.060	0.93	0.0050
V_max11_	1.00		0.28	0.190	1.03	0.0200
K_m11Suc_	100.00	NI	21.43	21.820	104.64	2.1200

NI - Non-identifiable parameter.

The mean and standard deviation are from 50 repetitions. For each of the iteration, the initial values for the parameters were initialized to random values in the range of 0 to 1, with the same initial values used for both cases.

**Table 4:** **Steady state analysis with actual parameters (from Rohwer) and with the estimated parameters without and with the informed prior**

	**Concentration (mmol/l)**		
**Species**	**From original parameters (Rohwer)**	**From estimated parameters**	
		**without**	**with**
		**Informed prior**	**Informed prior**
Fru	40.5800	44.6964	42.6673
Glc	30.1100	29.5312	29.7667
HexP	2.9850	2.6517	2.7880
Suc6P	0.0040	0.0054	0.0051
Suc	10.4130	10.7087	10.4975
Sucvac	0.0000	0.0000	0.0000
Glycolysis	0.0000	0.0000	0.0000
Phos	5.1000	5.1000	5.1000
UDP	0.2000	0.2000	0.2000
ADP	0.2000	0.2000	0.2000
ATP	1.0000	1.0000	1.0000
Glcex	5.0000	5.0000	5.0000
Fruex	5.0000	5.0000	5.0000

### Experiment 2: The gene regulatory network

**Figure 7:**
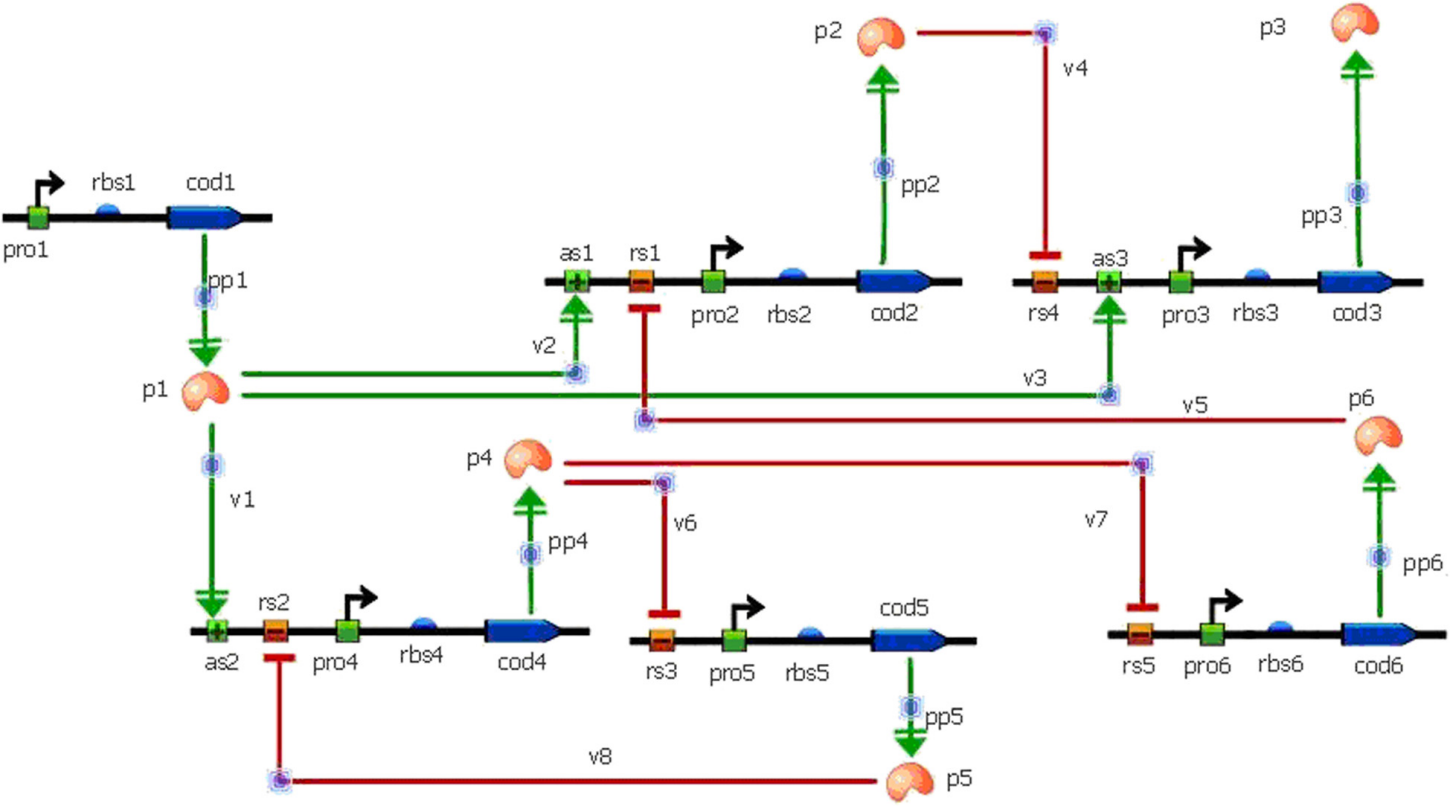
**Schematic diagram of the gene regulatory network reworked from the model provided by the Dream 6 Parameter estimation challenge.** The transcription and the translation are shown in a simplified form, combined as protein production. The symbols are, as: activator binding site, rs: repressor binding site, rbs: ribosomal binding site, pro: promoter, cod: protein coding region, p: protein.

In order to illustrate the broader applicability of this parameter estimation framework to general biological networks, the framework utilizing the informed prior was applied to a gene regulatory network, Figure 7. This experiment was based on the Dream6 challenge for the estimation of non-identifiable parameters in a predetermined model [43]. This model uses linear kinetics for mRNA degradation and protein synthesis and degradation. In addition, Hill type kinetics is used to model mRNA synthesis with one or two regulatory inputs. Each regulatory input works as either an activating or an inhibitory input. In the absence of a regulatory input to a gene, a constant rate of transcription is assumed. Both the network topology (Figure 7) and its mathematical description (Additional file 1) were provided by the contest. Protein production was modeled in combination with the transcription and translation steps.

A limited amount of microarray time-course data for all mRNA concentrations is initially provided for the wild-type variety. To reflect the actual scientific practice, additional time-course data of mRNA and protein concentrations in response to different network perturbations, in particular gene deletion, siRNA-mediated knock-down and change of RBS activity could be purchased within a predetermined budget.

The model has a total of 30 parameters from which 29 are to be estimated. The mRNA degradation rate is kept fixed to a nominal value of one. To test this framework, time-series data for the mRNAs and protein abundance for both the wild type and a mutant with RBS4 activity increased by 100%, were used. The main objective of this experiment is to determine a unique solution utilizing the CSUKF with an informed prior, despite non-identifiability and within the constraints of the provided data.

### Experimental setup

The experimental data that is available is a time-series over the interval of 0 to 20 seconds with a step size of 1 second. The lower bound of the constraint for CSUKF is set to 10^−8^ to ensure that the parameters are always positive. The upper bound is set at 100 as most parameters were in agreement with this value as reported in [55]. However, if any parameters would tend to approach this limit it could be raised and the estimation repeated. The experiment is divided into two phases. In the first phase, the mutant data with high RBS4 activity is used. The prior distribution of the model parameters are specified based on their ranking. The informed prior for the second phase of the experiment is thus formed based on the estimated parameter values and covariance matrix from the first phase of experiment. The second phase is then carried out with the wild type data. A synthetic noisy data set is provided by the contest. The noise model used by the contest is *y*_*noisy*_ = max[0, *y* + 0.1 × *r*_1_ + *C* × *r*_2_ × *y*], where y is the simulated value, r_1_ and r_2_ are Gaussian random variables with standard deviation of one and C = 0.2.

### First phase experiment

In the first stage the rank of the parameters is calculated. Having no information on the state probability distribution at the beginning of the experiment, the diagonal of both the state-estimation covariance matrix, *P*, and process noise covariance matrix, *Q*, are initialized with small random numbers between 0.001 and 0.1. The measurement noise covariance matrix *R* is initialized according to the noise model of the synthetic measurement data. During the second stage of the experiment both *P* and *Q* are initialized based on the ranking information derived from the first stage. Table 5 lists the ranking of the parameters along with the corresponding standard deviations used to formulate the informed prior. The ranking and standard deviation used to formulate the informed prior are mentioned in Additional file 1: Table S5.

**Table 5:** **Estimation result and standard deviation of the 29 parameters with and without informed priors**

		**Without informed prior**		**With informed prior**	
**Parameter name**	**Actual value**	**Mean**	**Std. Dev.**	**Mean**	**Std. Dev.**
p_degradation_rate	0.8	0.72	0.27	0.85	0.05
rbs1_strength	3.9	3.33	1.41	3.98	0.23
rbs2_strength	5.0	4.82	2.18	5.94	0.33
rbs3_strength	5.0	4.31	1.56	5.13	0.32
rbs4_strength	1.0	1.29	0.81	1.46	0.29
rbs5_strength	5.0	3.77	1.56	5.23	0.31
rbs6_strength	5.0	4.55	1.64	5.03	0.28
pro1_strength	3.0	2.94	0.15	3.04	0.05
pro2_strength	8.0	6.66	2.58	5.85	0.47
pro3_strength	6.0	9.14	4.43	7.12	0.68
pro4_strength	8.0	1.50	1.87	2.93	1.78
pro5_strength	3.0	3.46	0.80	3.03	0.07
pro6_strength	3.0	3.27	0.54	3.27	0.03
v1_Kd	1.0	1.40	1.62	1.54	0.18
v1_h	4.0	2.98	2.36	2.54	0.92
v2_Kd	1.0	1.17	0.74	1.87	0.15
v2_h	2.0	3.32	2.09	3.74	1.28
v3_Kd	0.1	0.61	0.31	0.56	0.18
v3_h	2.0	2.99	2.20	4.05	0.34
v4_Kd	10.0	7.17	3.10	8.04	1.12
v4_h	4.0	2.97	2.06	2.49	0.42
v5_Kd	1.0	2.16	1.52	2.22	0.41
v5_h	1.0	1.27	0.29	1.20	0.08
v6_Kd	0.1	0.64	0.57	0.28	0.02
v6_h	2.0	5.55	3.07	3.20	0.39
v7_Kd	0.1	0.48	0.28	0.26	0.02
v7_h	2.0	5.34	3.03	2.78	0.35
v8_Kd	0.2	2.14	2.40	0.41	0.30
v8_h	4.0	1.12	0.50	1.77	0.33

The mean and standard deviations are from 50 runs. For each run the parameters were initialized to small random values between 0 and 1. The same set of initial values was used for both cases, with and without the informed prior.

### Second phase experiment

The estimated parameter value and covariance matrix from the first phase experiment was used to formulate the informed prior for the second phase of the estimation. The parameters are initialized with small perturbations to the mean values of the first phase experiment. The matrix V is initialized to the final value of V from the first phase experiment. The process noise covariance matrix Q is initialized with the same matrix formulated with ranking used in the first phase. The measurement noise covariance matrix R is based on the same noise model as the experiment.

The results of the parameter estimation performed both with and without the informed prior are summarized in Table 5. The results present the mean and standard deviation from 50 repetitions of the experiment.

In Table 5 it was found that the estimated values more closely approached the actual values when using the informed prior, even after random initialization. This indicates higher estimation accuracy when using the informed prior compared to the estimation accuracy without informed prior. Furthermore the use of the informed prior allows for a very simple approach to making use of multiple data sets, i.e., the mutant in conjunction with the wild type, whereas to utilize two or more data sets without the informed prior requires parallel models subject to relationship constraints between the appropriate parameters. Undoubtedly the use of the second, mutant, data set in an essentially independent manner contributes to the improvement in accuracy. Additionally, the estimations conducted using the informed priors were more concise, as indicated by the low standard deviations, with the maximum relative standard deviation (parameter pro4_strength being just 60% of the mean value) compared to the estimation with no informed priors, where maximum standard deviation for 3 parameters exceed 100% of the mean value. In other words, the use of the informed prior for CSUFK is better apt to produce unique parameter estimation of a kinetic model, when presented with otherwise unidentifiable parameters.

## Discussion

Among the different types of mathematical models, kinetic modeling provides the most detailed picture of the working mechanism of a biological species. Despite this enormous prospect, the use of kinetic models has been limited, mostly due to it dependency on parameter values. The lack of accurate information on these parameter values from wet lab experiments derails the successful use of such models. In recent years the development of computational methods to estimate these parameters has been of great interest. However, most conventional methods do not guarantee an optimal solution and often fail to arrive at a satisfactory solution. To further complicate the estimation process, many parameters may be non-identifiable, i.e., parameters for which a unique solution of the values is not possible for a given model and available measurement data. The main objective of this work is to propose a complete parameter estimation framework that can handle these complexities of parameter estimation more effectively than the conventional methods.

This framework is composed of two interconnected modules, the parameter estimation module paired with an identifiability analysis (IA) module. We conducted two experiments to show the power of the proposed framework. In the first experiment each of the components of the IA module are utilized first to analyze and then to systematically solve the non-identifiability in the published model. The orthogonal ranking method was shown to be inadequate for properly locating non-identifiable parameters. This method was only able to identify three, of what turned out to be eight, non-identifiable parameters – K_i4F6P,_ K_i6F6P and_ K_m11Suc_ (Table 1). This was further evident by the inflated deviations found after running the parameter estimation with just these three parameters treated as being measured (Table 1). However the ranking scheme provided useful information towards solving the problem once fully identified. The IA module then utilizes a profile likelihood based analysis to more fully identify and furthermore to classify the non-identifiable parameters (Table 1), as either structurally or practically non-identifiable. Together these two techniques provide a clearer picture of the scale of the problem, with two thirds of the parameters being non-identifiable given the available measurement data. They also provide some guidance towards the possible cause of the problems and thus the solutions.

The solution begins by targeting the two structurally non-identifiable parameters, K_m6Suc6P and_ K_m6UDP_. The mean optimal transformation approach was used to determine any functional relationships between the parameters. This approach fits well into the framework as it can make use of the profile likelihood estimation data. Both of these parameters were found to have functional relationships to other practically non-identifiable parameters, in particular both were related to V_max6r_. Combined with the previous ranking data (ranked first) the framework was able to target this parameter as being crucial to solve the identifiability problems with this model. It should be noted that during this phase of the experiment there only constraint to solving the non-identifiability was keeping a fixed model. Thus any parameter may be targeted for measurement. One of the other benefits of functional analysis is to provide choices in the case where some parameters may be measurable or the model may be simplified. However, that still left several practically non-identifiable parameters to be dealt with.

A second method was used to determine correlation between parameters, based on the integrated parameter estimation algorithm. The mean and square root of the covariance is provided at each iteration of the CSUKF, which yields the correlation coefficient matrix. The correlation analysis identified strong between K_i3G6P_ and K_i4F6P_, and between K_i6F6P_, V_max6r_ and K_i6UDPGlc_. Typically the ranking would be used to select between parameters in the first correlation, however as K_i4F6P_ was not ranked (i.e., it was determined to be non-identifiable by the ranking algorithm) the framework selected it as a target for solution. In the latter relationship there already exists one parameter targeted for measurement, V_max6r_. However, after applying the various solutions, and using a measured value for V_max6r_, the non-identifiability persisted. The framework then identified the next parameter to target for measurement, similar to K_i4F6P_, K_i6F6P_ was selected as being found non-identifiable during ranking.

No functional relationship was found for the last two non-identifiable parameters, K_m11Suc_ and K_i6Suc6P_, so the last approach to solving the non-identifiability was applied, state trajectory analysis. Of the two, only K_m11Suc_ displayed large variations in its state trajectories with fructose and sucrose (Figure 5). Large variations in the state trajectories are indicative of points of more uncertainty. Thus the framework identified that additional (and/or more accurate) measurement data at this point for the fructose and sucrose states may solve the non-identifiability in K_m11Suc_.

With no additional information available to provide a solution for K_i6Suc6P_ the framework provides no direct solution. However, after applying the existing solutions, measuring V_max6r_, K_i6F6P_ and K_i4F6P_, and doubling the measurements of fructose and sucrose (at the same accuracy), the IA determined that all remaining parameters were identifiable. One possible reason is an as yet undetermined functional relationship between K_i6Suc6P_ and one or more other parameters.

By creating an integrated framework that combines several interrelated techniques it was possible to not only correctly analyze the non-identifiability but to solve it, requiring no more measured parameters than originally required by the ranking algorithm alone. However, despite selecting two of the three parameters originally identified during ranking, the correct identification of the third was crucial to correctly estimating the parameters (Table 2) and recreating the state trajectories of the original model (Figure 6).

As previously mentioned, in evaluating the IA no constraints were placed on the acquisition of additional data. To complete the evaluation the most stringent case was considered, where it is not possible to measure any of the unknown parameters, to obtain additional measurement data or to make any changes to the model. In this case, the IA is used not to formulate suggested solutions to non-identifiability, but instead it is used to formulate the informed prior. The results (Table 3) clearly show the advantage of using a parameter estimation technique based on the Bayesian approach. The inclusion of the informed prior to initialize the filtering technique, which sets the uncertainty of the parameter instead of a random initialization, leads to a unique estimation value for nearly all of the parameters, even in the presence of non-identifiability.

The second experiment is used to further validate the use of the informed prior for improved parameter estimation. The model, a gene regulatory network, comes from a different area of biological research. The results are similar to the first experiment, a unique estimation of value for nearly all of the parameters, even in the presence of non-identifiability. What is more interesting is the manner in which the experiment proceeds, making use of two data sets for different genetic cases (the wild type and a mutant with upregulated RBS4) as opposed to increased frequency of measurement. Essentially the Bayesian approach of constantly refining the prediction allows for multiple data sets to be used sequentially. That is, the estimated parameter values and final covariance matrix from the first data set, may be used to initialize the informed prior for the second data set. Contrast this to a least squares global optimizer which benefits less from this refinement preferring the parallel model approach. Thus, this approach is both conceptually and computationally more efficient when utilizing parallel data sets, which is the most likely method for increased measurement data in biological systems.

## Conclusion

The widespread adoption of modeling techniques to biological problems is driving the need for parameter estimation methods adapted to the inherent limitations of the field. The highly non-linear and dynamic nature of biological systems combined with the often severely limited and noisy measurement data is further complicated by the issue of non-identifiability.

The unified parameter estimation framework presented here provides a robust and complete solution by coupling parameter estimation and identifiability analysis. The parameter estimation makes use of the recently proposed constrained square-root unscented Kalman filter, designed specifically to address the estimation problem in biological modeling. The identifiability module includes multiple approaches, which may be further extended, to identify, classify and suggest solutions for non-identifiable parameters. By leveraging the unique properties of the CSUKF, the unified framework is also able to provide an informed prior for parameter estimation, when non-identifiability cannot be directly solved.

The results from applying this framework show that these tools combine to yield reliable and unique estimations, even when constrained by limited and noisy measurement data.

## Availability and requirements

The software is available upon request from the author Syed M. Baker.

- **Project name:** Unified framework for parameter estimation
- **Operating system(s):** Platform independent
- **Programming language:** MATLAB
- **Other requirements:** None
- **License:** GNU GPL
- **Any restrictions to use by non-academics:** None

### Endnote

^a^The SBML model is called model1.sbml in the folder:

DREAM6_ParEst_Data_v4\Model1\Model Representations.

## Additional file

Additional file 1:
**A supplement is provided with supporting information.** **Supplement 1.** Provides detailed background information describing the method of orthogonal based ranking. **Supplement 2.** Gives the specific rate laws used in the sugarcane culm model. **Supplement 3.** Provides additional illustrations and discussion to more fully explain the non-identifiability issue due to functional relationships. In **supplement 4.** The specific rate laws of the Gene regulatory network are provided. **Supplement 5.** Has a table of the complete set of results from the gene regulatory network model. Lastly **Supplements 6** and **7.** Provide verification tables comparing the state results from the original SBML models in Copasi to the models in MATLAB after importing the SBML files. **Supplement 6.** Is for the sugarcane model and **Supplement 7.** For the gene regulatory network.

## Abbreviations

ACE: Alternating conditional expectation
CSUKF: Constrained square-root unscented Kalman filter
FIM: Fisher information matrix
EKF: Extended Kalman filter
IA: Identifiability analysis
KF: Kalman filtering
ML: Maximum likelihood
MLE: Maximum likelihood estimation
MOTA: Mean optimal transformation approach
ODE: Ordinary differential equation
SMC: Sequential Monte Carlo
SR-UKF: Square-root unscented Kalman filter
UKF: Unscented Kalman filter

## References

[1] Klipp E, Herwig R, Kowald A, Wierling C, Lehrach H (2005). Systems biology in practice: concepts, implementation and application.

[2] Borger S, Liebermeister W, Klipp E (2006). Prediction of enzyme kinetic parameters based on statistical learning. Genome Inform.

[3] Sun X, Jin L, Xiong M (2008). Extended kalman filter for estimation of parameters in nonlinear state-space models of biochemical networks. PLoS One.

[4] Liu X, Niranjan M (2012). State and parameter estimation of the heat shock response system using Kalman and particle filters. Bioinformatics.

[5] Doucet AaDF, Nando and Gordon, Neil. Sequential Monte Carlo methods in practice. New York: Springer; 2001.

[6] Nakamura K, Yoshida R, Nagasaki M, Miyano S, Higuchi T (2009). Parameter estimation of in silico biological pathways with particle filtering towards a petascale computing. Pac Symp Biocomput.

[7] Qiang Bo WZ-Z. Application of Unscented Particle Filtering for Estimating Parameters and Hidden Variables in Gene Regulatory Network. In: 4th International Conference on Bioinformatics and Biomedical Engineering (iCBBE); Chengdu. 2010.

[8] Julier SJ, Uhlmann JK (1997). A new extension of the Kalman Filter to nonlinear systems.

[9] Quach M, Brunel N, D’Alché-Buc F (2007). Estimating parameters and hidden variables in non-linear state-space models based on ODEs for biological networks inference. Bioinformatics.

[10] Julier SJ, Uhlmann JK (2004). Unscented Filtering and Nonlinear Estimation.

[11] Welch G, Bishop G (1995). An Introduction to the Kalman Filter.

[12] Lillacci G, Khammash M (2010). Parameter Estimation and Model Selection in Computational Biology. PLoS Comput Biol.

[13] Al-Hussein A, Haldar A. A comparison of unscented and extended Kalman filtering for nonlinear system identification. In: 12^th^ International Conference on Applications of Statistics and Probability in Civil Engineering. Vancouver, B.C.; 2015.

[14] Leven WF, Lanterman AD. Multiple Target Tracking with Symmetric Measurement Equations Using Unscented Kalman and Particle Filters. In Proceedings of the 36^th^ Southeastern Symposium on System Theory; 2004.

[15] Wan E, Merwe RVD. Chapter 7 The Unscented Kalman Filter. In: 2001. Wiley: 221-280.

[16] Vachhani P, Narasimhan S, Rengaswamy R (2006). Robust and reliable estimation via Unscented Recursive Nonlinear Dynamic Data Reconciliation. J Process Control.

[17] Murtuza Baker S, Poskar CH, Schreiber F, Junker BH (2013). An improved constraint filtering technique for inferring hidden states and parameters of a biological model. Bioinformatics.

[18] Chis O-T, Banga JR, Balsa-Canto E. Structural Identifiability of Systems Biology Models: A Critical Comparison of Methods. PLoS ONE. 2011;6(11):e27755 10.1371/journal.pone.0027755 PMC3222653 22132135

[19] Raue A, Kreutz C, Maiwald T, Klingmuller U, Timmer J (2011). Addressing parameter identifiability by model-based experimentation. IET Syst Biol.

[20] Raue A, Becker V, Klingmuller U, Timmer J (2010). Identifiability and observability analysis for experimental design in nonlinear dynamical models. Chaos.

[21] Raue A, Kreutz C, Maiwald T, Bachmann J, Schilling M, Klingmüller U (2009). Structural and practical identifiability analysis of partially observed dynamical models by exploiting the profile likelihood. Bioinformatics.

[22] Jazwinski AH. Stochastic Processes and Filtering Theory, Vol. 6: Academic Press. 1970.

[23] Sitz A, Schwarz U, Kurths J, Voss HU (2002). Estimation of parameters and unobserved components for nonlinear systems from noisy time series. Phys Rev E.

[24] Quaiser T, Monnigmann M (2009). Systematic identifiability testing for unambiguous mechanistic modeling–application to JAK-STAT, MAP kinase, and NF-kappaB signaling pathway models. BMC Syst Biol.

[25] Cobelli C, DiStefano JJ (1980). Parameter and structural identifiability concepts and ambiguities: a critical review and analysis. Am J Physiol Regul Integr Comp Physiol.

[26] Yao KZ, Shaw BM, Kou B, McAuley KB, Bacon DW (2003). Modeling Ethylene/Butene Copolymerization with Multi-site Catalysts: Parameter Estimability and Experimental Design. Polym React Eng.

[27] Chis O, Banga JR, Balsa-Canto E (2011). GenSSI: a software toolbox for structural identifiability analysis of biological models. Bioinformatics.

[28] Samaniego FJ (2010). A Comparison of the Bayesian and Frequentist Approaches to Estimation, vol. 6.

[29] McAuley KB, Wu S, Harris TJ. Selecting Parameters to Estimate to Obtain the Best Model Predictions Proceedings of the 2010 International Conference on Modelling, Identification and Control, Okayama, Japan, July 17-19, 2010.

[30] Antoniewicz MR, Stephanopoulos G, Kelleher JK (2006). Evaluation of regression models in metabolic physiology: predicting fluxes from isotopic data without knowledge of the pathway. Metabolomics.

[31] Venzon DJ, Moolgavkar SH (1988). A Method for Computing Profile-Likelihood Based Confidence Intervals. Appl Stat.

[32] Neale MC, Miller MB (1997). The Use of Likelihood-Based Confidence Intervals in Genetic Models.. Behavior Genetics.

[33] Thacker NA, Lacey AJ. Tutorial: The Kalman Filter. In: Imaging Science and Biomedical Engineering Division, Medical School, University of Manchester. TiNA; 1998.

[34] Hengl S, Kreutz C, Timmer J, Maiwald T (2007). Data-based identifiability analysis of non-linear dynamical models. Bioinformatics.

[35] Kay SM. Fundamentals of statistical signal processing: estimation theory. Prentice-Hall, Inc. 1993.

[36] Schenkendorf R, Kremling A, Mangold M (2009). Optimal Experimental Design with the Sigma Point method.

[37] Breiman L, Friedman JH. Estimating Optimal Transformations for Multiple Regression and Correlation: Rejoinder. Journal of the American Statistical Association. 1985;80:614-619. doi:10.2307/2288477.

[38] Neath AA, Samaniego FJ (1997). On the Efficacy of Bayesian Inference for Nonidentifiable Models. Am Stat.

[39] Rannala B (2002). Identifiability of parameters in MCMC Bayesian inference of phylogeny. Syst Biol.

[40] Rohwer JM, Botha FC (2001). Analysis of sucrose accumulation in the sugar cane culm on the basis of in vitro kinetic data. Biochem J.

[41] Uys L, Botha FC, Hofmeyr JHS, Rohwer JM (2007). Kinetic model of sucrose accumulation in maturing sugarcane culm tissue. Phytochemistry.

[42] Sugarcane model file from Rohwer and Botha 2001 - SBML Model [http://www.ebi.ac.uk/biomodels-main/BIOMD0000000023]

[43] Prill RJ, Daniel M, Julio S-R, Sorger PK, Alexopoulos LG, Xiaowei X (2010). Towards a Rigorous Assessment of Systems Biology Models: The DREAM3 Challenges. PLoS One.

[44] DREAM6 Estimation of Model Parameters Challenge - SBML Model [https://www.synapse.org/#!Synapse:syn2843038]

[45] Chen WW, Niepel M, Sorger PK. Classic and contemporary approaches to modeling biochemical reactions. Genes & development. 2010;24:1861-1875. doi:10.1101/gad.1945410. 10.1101/gad.1945410 PMC2932968 20810646

[46] Berit FL, Bjarne AF. Parameter ranking by orthogonalization Applied to nonlinear mechanistic models. Automatica. 2008;44:278-281.

[47] Miao H, Xia X, Perelson AS, Wu H. On Identifiability of Nonlinear ODE Models and Applications in Viral Dynamics. SIAM Review. 2011;53(1):3-39. 10.1137/090757009 PMC3140286 21785515

[48] Faller D, Klingmüller U, Timmer J. Simulation Methods for Optimal Experimental Design in Systems Biology. SIMULATION. 2003;79:717-725.

[49] Rodriguez-Fernandez M, Mendes P, Banga JR (2006). A hybrid approach for efficient and robust parameter estimation in biochemical pathways. Bio Systems.

[50] Guedj J, Thiebaut R, Commenges D. Practical identifiability of HIV dynamics models. Bull Math Biol. 2007;69:2493-2513. doi:10.1007/s11538-007-9228-7. 10.1007/s11538-007-9228-7 17557186

[51] Achcar F, Kerkhoven EJ, Bakker BM, Barrett MP, Breitling R (2012). Dynamic modelling under uncertainty: the case of Trypanosoma brucei energy metabolism. PLoS Comput Biol.

[52] Jia G, Stephanopoulos GN, Gunawan R. Parameter estimation of kinetic models from metabolic profiles: two-phase dynamic decoupling method. Bioinformatics. 2011;27:1964-1970. 10.1093/bioinformatics/btr293 PMC6281308 21558155

[53] Suzdaleva E (2007). Initial conditions for Kalman filtering: prior knowledge specification.

[54] Lindley DV, El-Sayyad GM. The Bayesian Estimation of a Linear Functional Relationships. Journal of the Royal Statistical Society. Series B (Methodological). 1968;30:190-202. doi:10.2307/2984471.

[55] Steiert B, Raue A, Timmer J, Kreutz C (2012). Experimental design for parameter estimation of gene regulatory networks. PLoS One.

