# Supplementary material for: A unified framework for estimating parameters of kinetic biological models
Source: BMC Bioinformatics. 2015 Mar 27;16:104. doi: 10.1186/s12859-015-0500-9 (PMC4464135; doi:10.1186/s12859-015-0500-9)
Supplement: Additional file 1: — A supplement is provided with supporting information. Supplement 1. Provides detailed background information describing the method of orthogonal based ranking. Supplement 2. Gives the specific rate laws used in the sugarcane culm model. Supplement 3. Provides additional illustrations and discussion to more fully explain the non-identifiability issue due to functional relationships. In supplement 4. The specific rate laws of the Gene regulatory network are provided. Supplement 5. Has a table of the complete set of results from the gene regulatory network model. Lastly Supplements 6 and 7. Provide verification tables comparing the state results from the original SBML models in Copasi to the models in MATLAB after importing the SBML files. Supplement 6. Is for the sugarcane model and Supplement 7. For the gene regulatory network. [file 12859_2015_500_MOESM1_ESM.pdf]

# Table of Contents


**Supplement 1: Orthogonal based ranking algorithm .................................................2**

**Supplement 2: The model ODEs and rate laws of the sugarcane culm model...........3**

**Supplement 3: Non-identifiability due to a functional relationship ...........................5**

**Supplement 4: The rate laws and model ODEs of the gene regulatory network.......7**

**Supplement 5: Gene Regulatory Network Model Results ........................................10**

**Supplement 6: MATLAB model verification of the sugarcane culm model............11**

**Supplement 7: MATLAB model verification of the gene regulatory network.........12**

## Supplement 1: Orthogonal based ranking algorithm

1. Calculate the sensitivity matrix $Z$ .
2. Calculate the sum of squared values of each column of $Z$ and choose the column with the highest value as the most estimable.
3. Add the chosen column to $E_L$ where $L=1$ for the first iteration. With increasing number of iterations the size of $E_L$ matrix grows as columns from $Z$ are added to $E_L$.
4. Calculate an orthogonal projection $\hat{Z}_L$ for the column that exhibits the highest independence to the vector space spanned by $E_L$

$$\hat{Z}_L = E_L(E_L^T E_L)^{-1} E_L^T Z \qquad (1)$$

5. As a measure of independence the residual matrix $Rs_L = Z - \hat{Z}_L$ is calculated.
6. The sum of squares value for each column of the $Rs_L$ matrix is calculated, resulting in the vector $Cs_L$. The column corresponding to the largest sum of squares is chosen for the next estimable parameter.
7. Select the corresponding column in $Z$ and augment it with the matrix $E_L$ by marking the new column.
8. Iterate steps 4-7 until the cut-off value is reached or until all the parameters have been ranked and selected to be identifiable.

## Supplement 2: The model ODEs and rate laws of the sugarcane culm model

The SBML model is available from the Biomodels database:

**http://www.ebi.ac.uk/biomodels-main/BIOMD0000000023**

The ODEs for the model are:

$$\frac{d[Glc]}{dt} = v_2 - v_3 + v_9$$

$$\frac{d[Fru]}{dt} = v_1 - v_4 - v_5 - v_8 + v_9$$

$$\frac{d[HexP]}{dt} = v_3 + v_4 + v_5 - 2v_6 - v_8 - v_{10}$$

$$\frac{d[Suc6P]}{dt} = v_6 - v_7$$

$$\frac{d[Suc]}{dt} = v_7 + v_8 - v_9 - v_{11}$$

Where the rate laws used in the Sugarcane calm model, as developed by Rohwer and Botha [40], are:

$$v_1 = V_{\max 1} \frac{[Fru_{ex}]}{k_{m1Fru,ex}\left(1 + \frac{[Fru]}{k_{i1Fru}}\right) + [Fru_{ex}]}$$

$$v_2 = V_{\max 2} \frac{[Glc_{ex}]}{k_{m2Glc,ex}\left(1 + \frac{[Glc]}{k_{i2Glc}}\right) + [Glc_{ex}]}$$

$$v_3 = V_{\max 3} \frac{\frac{[Glc]}{k_{m3Glc}} \frac{[ATP]}{k_{m3ATP}}}{\left(1 + \frac{[ATP]}{k_{m3ATP}}\right) \cdot \left(1 + \frac{[Glc]}{k_{m3Glc}} + \frac{[Fru]}{k_{m4Fru}} + \frac{0.113[HexP]}{k_{i3Glc6P}} + \frac{0.0575[HexP]}{k_{i4Fru6P}}\right)}$$

$$v_4 = V_{\max 4} \frac{\frac{[Fru]}{k_{m4Fru}} \frac{[ATP]}{k_{m4ATP}}}{\left(1 + \frac{[ATP]}{k_{m4ATP}}\right) \cdot \left(1 + \frac{[Glc]}{k_{m3Glc}} + \frac{[Fru]}{k_{m4Fru}} + \frac{0.113[HexP]}{k_{i3Glc6P}} + \frac{0.0575[HexP]}{k_{i4Fru6P}}\right)}$$

$$v_5 = V_{\max 5} \frac{\frac{[Fru]}{k_{m5Fru}} \frac{[ATP]}{k_{m5ATP}}}{\left(1 + \frac{[Fru]}{k_{i5Fru}}\right) \cdot \left(1 + \frac{[Fru]}{k_{m5Fru}} + \frac{[ATP]}{k_{m5ATP}} + \frac{[Fru][ATP]}{k_{m5Fru} \cdot k_{m5ATP}} + \frac{[ADP]}{k_{i5ADP}}\right)}$$

$$v_6 = V_6^f \left( \frac{(0.0575[HexP])(0.8231[HexP]) - \frac{[Suc][UDP]}{k_{eq6}}}{(0.0575[HexP])(0.8231[HexP]) \cdot \left(1 + \frac{[Suc6P]}{k_{i6Suc6P}}\right) + k_{m6Fru6P}(0.8231[HexP] + k_{i6UDPGlc}) \cdot \left(1 + \frac{[P]}{k_{i6P}}\right) + k_{m6UDPGlc}(0.0575[HexP]) \cdot \left(\frac{V_6^f}{V_6^r k_{eq6}}\right)} \right) \cdot \left( k_{m6UDP}[Suc6P] \cdot \left(1 + \frac{0.8231[HexP]}{k_{i6UDPGlc}}\right) + k_{m6Suc6P}[UDP] \cdot \left(1 + \frac{k_{m6UDPGlc}(0.0575[HexP])}{k_{i6UDPGlc} k_{m6Fru6P}\left(1 + \frac{[P]}{k_{i6P}}\right)}\right) + [Suc6P] \cdot \left(1 + \frac{0.0575[HexP]}{k_{i6Fru6P}}\right) \right)$$

$$v_7 = V_{\max 7} \frac{[Suc6P]}{k_{m7Suc6P} + [Suc6P]}$$

$$v_8 = -V_8^f \frac{[Suc][UDP] - \left(\frac{0.8231[HexP] \cdot [Fru]}{k_{eq8}}\right)}{[Suc][UDP]\left(1 + \frac{[Fru]}{k_{i8Fru}}\right) + k_{m8Suc}(k_{i8UDP} + [UDP]) + k_{m8UDP}[Suc] + k_{m8UDPGlc}[Fru] \cdot \left(1 + \frac{[UDP]}{k_{i8UDP}}\right) \cdot \left(\frac{V_8^f}{V_8^r k_{eq8}}\right) + k_{m8Fru}(0.8231[HexP]) \cdot \left(1 + \frac{k_{m8UDP} k_{m8Suc}[Suc]}{k_{i7UDP}}\right) + [Fru] \cdot \left(1 + \frac{[Suc]}{k_{i8Suc}}\right)}$$

$$v_9 = V_{\max 9} \frac{[Suc]}{\left(1 + \frac{[Glc]}{k_{i9Glc}}\right) \cdot \left(k_{m9Suc}\left(1 + \frac{[Fru]}{k_{i9Fru}}\right) + [Suc]\right)}$$

$$v_{10} = V_{\max 10} \frac{0.0575[HexP]}{k_{m10Fru6P} + 0.0575[HexP]}$$

$$v_{11} = V_{\max 11} \frac{[Suc]}{k_{m11Suc} + [Suc]}$$

# Supplement 3: Non-identifiability due to a functional relationship

The orthogonal identifiability analysis of the ranking method determined that the three parameters, $K_{i4F6P}$, $K_{i6F6P}$ and $K_{m11Suc}$, were non-identifiable. In order to determine if any functional relationships exist with the non-identifiable parameters an exhaustive functional analysis of these parameters was carried out. This analysis reveals a strong linear relationship between $K_{m11Suc}$ and $V_{\max 11}$ as shown in the Supplementary Figure 1a. A second nonlinear (hyperbolic) relationship was found between $K_{i3G6P}$ and $K_{i4F6P}$, see Supplementary Figure 1b. No functional relationship was found between the last of the non-identifiable parameters, $K_{i6F6P}$, and any of the identifiable parameters. One method for dealing with non-identifiable parameters is to measure them directly. Although not typically possible in biological systems, the actual values are available for this model. Thus the estimation process was repeated using the actual values of the non-identifiable parameters while estimating the identifiable ones. The results of the estimation remain mixed, with roughly an equal number of parameters showing improvement and deterioration. Steady state analysis with the estimated parameters from Table 2 found the same stable steady state as was found with the actual parameter values (Supplementary Table 1). This confirms that although some of the parameters are non-identifiable the estimated parameter values remain valid for describing the actual dynamics of the system.

There is a clear indication that among the 9 identifiable parameters from the sensitivity method, some are still non-identifiable.

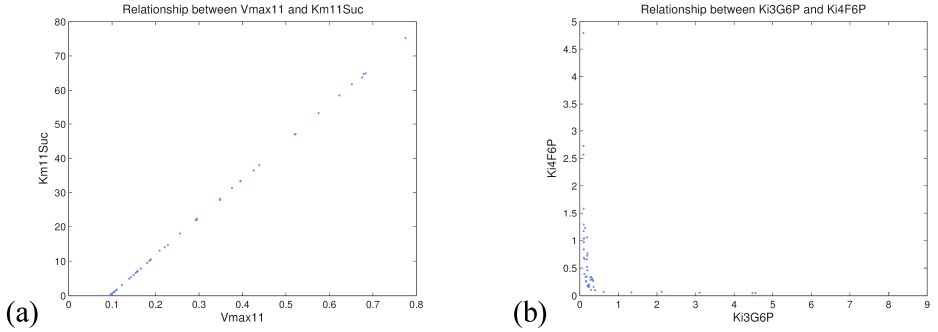


Supplementary Figure 1: Functional relationships between parameters. a) Linear; the relationship between $K_{m11Suc}$ and $V_{\max 11}$. b) near hyperbolic; the relationship between $K_{i3G6P}$ and $K_{i4F6P}$.

| Metabolite | Concentration | |
|---|---|---|
| | Actual Parameter | Estimated Parameter |
| Fru | 1.00 | 1.00 |
| Glc | 1.00 | 1.00 |
| HexP | 0.10 | 0.67 |
| HexP | 10.00 | 0.63 |
| Suc6P | 0.07 | 0.45 |
| Suc | 1.40 | 0.32 |
| Sucvac | 0.20 | 0.34 |
| Glycolysis | 0.30 | 4.73 |
| Phos | 0.10 | 5.97 |
| UDP | 0.40 | 0.65 |
| ADP | 1.00 | 0.28 |
| ATP | 100.00 | 21.43 |
| Glcex | 100.00 | 21.43 |
| Fruex | 100.00 | 21.43 |

Supplementary Table 1: Steady state metabolite concentration as calculated using the actual parameter set from Rohwer and Botha [40] and with the estimated parameters.

# Supplement 4: The rate laws and model ODEs of the gene regulatory network

The SBML model is available from the Sage Bionetworks' Synapse database:

**https://www.synapse.org/#!Synapse:syn2843038**

The model file is **model1.sbml** in the folder:

**DREAM6_ParEst_Data_v4\Model1\Model Representations**

The rate laws of the model are:

$$r_1 = pro1\_strength$$

$$r_2 = pp1\_mrna\_degradation\_rate \times pp1\_mrna$$

$$r_3 = rbs1\_strength \times pp1\_mrna$$

$$r_4 = p1\_degradation\_rate \times p1$$

$$r_5 = pro2\_strength \times as1 \times rs1$$

$$r_6 = pp2\_mrna\_degradation\_rate \times pp2\_mrna$$

$$r_7 = rbs2\_strength \times pp2\_mrna$$

$$r_8 = p2\_degradation\_rate \times p2$$

$$r_9 = pro3\_strength \times as3 \times rs4$$

$$r_{10} = pp3\_mrna\_degradation\_rate \times pp3\_mrna$$

$$r_{11} = rbs3\_strength \times pp3\_mrna$$

$$r_{12} = p3\_degradation\_rate \times p3$$

$$r_{13} = pro4\_strength \times as2 \times rs2$$

$$r_{14} = pp4\_mrna\_degradation\_rate \times pp4\_mrna$$

$$r_{15} = rbs4\_strength \times pp4\_mrna$$

$$r_{16} = p4\_degradation\_rate \times p4$$

$$r_{17} = pro5\_strength \times rs3$$

$$r_{18} = pp5\_mrna\_degradation\_rate \times pp5\_mrna$$

$$r_{19} = rbs5\_strength \times pp5\_mrna$$

$$r_{20} = p5\_degradation\_rate \times p5$$

$$r_{21} = pro6\_strength \times rs5$$

$$r_{22} = pp6\_mrna\_\deg radation\_rate \times pp6\_mrna$$

$$r_{23} = rbs6\_strength \times pp6\_mrna$$

$$r_{24} = p6\_\deg radation\_rate \times p6$$

$$as_1 = \frac{\left(\frac{p1}{v2\_kd}\right)^{v2\_h}}{\left(1+\frac{p1}{v2\_kd}\right)^{v2\_h}}$$

$$as_2 = \frac{\left(\frac{p1}{v1\_kd}\right)^{v1\_h}}{\left(1+\frac{p1}{v1\_kd}\right)^{v1\_h}}$$

$$as_3 = \frac{\left(\frac{p1}{v3\_kd}\right)^{v3\_h}}{\left(1+\frac{p1}{v3\_kd}\right)^{v3\_h}}$$

$$rs_1 = \frac{1}{1+\left(\frac{p6}{v5\_kd}\right)^{v5\_h}}$$

$$rs_2 = \frac{1}{1+\left(\frac{p5}{v8\_kd}\right)^{v8\_h}}$$

$$rs_3 = \frac{1}{1+\left(\frac{p4}{v6\_kd}\right)^{v6\_h}}$$

$$rs_4 = \frac{1}{1+\left(\frac{p2}{v4\_kd}\right)^{v4\_h}}$$

$$rs_5 = \frac{1}{1+\left(\frac{p4}{v7\_kd}\right)^{v7\_h}}$$

The naming of the variables are, $as$: activator binding site, $rs$: repression binding site, $rbs$: ribosomal binding site strength, $pro$: promoter strength, $p$: protein concentration, $pp$: protein production process.

The ODEs of the model are:

$$\frac{d(pp1\_mrna)}{dt} = r_1 - r_2$$

$$\frac{d(p1)}{dt} = r_3 - r_4$$

$$\frac{d(pp2\_mrna)}{dt} = r_5 - r_6$$

$$\frac{d(p2)}{dt} = r_7 - r_8$$

$$\frac{d(pp3\_mrna)}{dt} = r_9 - r_{10}$$

$$\frac{d(p3)}{dt} = r_{11} - r_{12}$$

$$\frac{d(pp4\_mrna)}{dt} = r_{13} - r_{14}$$

$$\frac{d(p4)}{dt} = r_{15} - r_{16}$$

$$\frac{d(pp5\_mrna)}{dt} = r_{17} - r_{18}$$

$$\frac{d(p5)}{dt} = r_{19} - r_{20}$$

$$\frac{d(pp6\_mrna)}{dt} = r_{21} - r_{22}$$

$$\frac{d(p6)}{dt} = r_{23} - r_{24}$$

# Supplement 5: Gene Regulatory Network Model Results

| Parameter Name | Ranking | Initialized Std. Dev. |
|---|---|---|
| rbs4_strength | 1 | 0.001 |
| pro6_strength | 2 | 0.002 |
| v8_h | 3 | 0.003 |
| pro1_strength | 4 | 0.004 |
| pro5_strength | 5 | 0.005 |
| pro3_strength | 6 | 0.006 |
| pro2_strength | 7 | 0.007 |
| pro4_strength | 8 | 0.008 |
| rbs6_strength | 9 | 0.009 |
| rbs5_strength | 10 | 0.010 |
| v5_h | 11 | 0.011 |
| v7_Kd | 12 | 0.012 |
| v6_Kd | 13 | 0.013 |
| p_degradation_rat | 14 | 0.014 |
| v4_h | 15 | 0.015 |
| v7_h | 16 | 0.016 |
| rbs1_strength | 17 | 0.017 |
| rbs3_strength | 18 | 0.018 |
| v6_h | 19 | 0.019 |
| v8_Kd | 20 | 0.020 |
| v3_h | 21 | 0.021 |
| rbs2_strength | 22 | 0.022 |
| v5_Kd | 23 | 0.023 |
| v2_h | 24 | 0.024 |
| v1_Kd | 25 | 0.025 |
| v2_Kd | 26 | 0.026 |
| v3_Kd | 27 | 0.027 |
| v1_h | 28 | 0.028 |
| v4_Kd | 29 | 0.029 |

Supplementary Table 5: Ranking and standard deviation of parameters used in formulating the informed prior. The variances are used to initialize the diagonal of the state-estimation covariance matrix, $P$, and the process noise covariance matrix, $Q$.

## Supplement 6: MATLAB model verification of the sugarcane culm model

The tables below shows the state values generated by the original SBML model in Copasi (Table 6a) and by the model implemented in MATLAB (Table 6b). In both cases the models are using the same set of initial parameter values.

| | | | Generated by Copasi | | |
|---|---|---|---|---|---|
| # Time | Fru | Glc | HexP | Suc6P | Suc |
| 0 | 1 | 1 | 1 | 1 | 1 |
| 1 | 1.1721 | 1.1624 | 1.2339 | 0.5617 | 1.3941 |
| 2 | 1.3508 | 1.3310 | 1.4591 | 0.1824 | 1.7201 |
| 3 | 1.5320 | 1.5021 | 1.6742 | 0.0099 | 1.8363 |
| 4 | 1.7094 | 1.6705 | 1.8797 | 0.0025 | 1.7924 |
| 5 | 1.8816 | 1.8349 | 2.0761 | 0.0028 | 1.7475 |
| 6 | 2.0487 | 1.9958 | 2.2634 | 0.0032 | 1.7091 |
| 7 | 2.2112 | 2.1533 | 2.4422 | 0.0036 | 1.6770 |
| 8 | 2.3694 | 2.3078 | 2.6126 | 0.0039 | 1.6507 |
| 9 | 2.5234 | 2.4594 | 2.7751 | 0.0043 | 1.6300 |
| 10 | 2.6735 | 2.6085 | 2.9298 | 0.0046 | 1.6145 |

Supplementary Table 6a: Results from Copasi using the original SBML model of the sugarcane culm tissue and the default set of initial parameter values.

| | | | Generated by MATLAB | | |
|---|---|---|---|---|---|
| Time | Fru | Glc | HexP | Suc6P | Suc |
| 0 | 1 | 1 | 1 | 1 | 1 |
| 1 | 1.1721 | 1.1624 | 1.2339 | 0.5617 | 1.3941 |
| 2 | 1.3508 | 1.3310 | 1.4591 | 0.1824 | 1.7201 |
| 3 | 1.5320 | 1.5021 | 1.6742 | 0.0099 | 1.8363 |
| 4 | 1.7094 | 1.6705 | 1.8797 | 0.0025 | 1.7924 |
| 5 | 1.8816 | 1.8349 | 2.0760 | 0.0028 | 1.7475 |
| 6 | 2.0487 | 1.9958 | 2.2634 | 0.0032 | 1.7091 |
| 7 | 2.2112 | 2.1533 | 2.4422 | 0.0036 | 1.6770 |
| 8 | 2.3694 | 2.3078 | 2.6126 | 0.0039 | 1.6507 |
| 9 | 2.5234 | 2.4594 | 2.7751 | 0.0043 | 1.6300 |
| 10 | 2.6735 | 2.6085 | 2.9298 | 0.0046 | 1.6145 |

Supplementary Table 6b: Results from Copasi using the original SBML model of the sugarcane culm tissue and the default set of initial parameter values.

## Supplement 7: MATLAB model verification of the gene regulatory network

The tables below shows the state values generated by the original SBML model in Copasi (Table 7a) and by the model implemented in MATLAB (Table 7b). In both cases the models are using the same set of initial parameter values.

| | | | | | | Generated by Copasi | | | | | | |
|---|---|---|---|---|---|---|---|---|---|---|---|---|
| # Time | pp1_mrna | p1 | pp2_mrna | p2 | pp3_mrna | p3 | pp4_mrna | p4 | pp5_mrna | p5 | pp6_mrna | p6 |
| 0 | 0 | 1 | 0 | 1 | 0 | 1 | 0 | 1 | 0 | 1 | 0 | 1 |
| 1 | 0.6321 | 0.6321 | 0.1580 | 0.4337 | 0.1622 | 0.4346 | 0.1580 | 0.4337 | 0.3950 | 0.5232 | 0.3950 | 0.5232 |
| 2 | 0.8647 | 0.7293 | 0.2271 | 0.2872 | 0.2518 | 0.2970 | 0.2271 | 0.2872 | 0.6204 | 0.5327 | 0.6204 | 0.5327 |
| 3 | 0.9502 | 0.8506 | 0.2605 | 0.2629 | 0.3145 | 0.2929 | 0.2605 | 0.2629 | 0.7266 | 0.6318 | 0.7266 | 0.6318 |
| 4 | 0.9817 | 0.9267 | 0.2739 | 0.2670 | 0.3523 | 0.3216 | 0.2739 | 0.2670 | 0.7671 | 0.7088 | 0.7671 | 0.7088 |
| 5 | 0.9933 | 0.9663 | 0.2784 | 0.2733 | 0.3722 | 0.3490 | 0.2784 | 0.2733 | 0.7796 | 0.7511 | 0.7796 | 0.7511 |
| 6 | 0.9975 | 0.9851 | 0.2797 | 0.2770 | 0.3820 | 0.3676 | 0.2797 | 0.2770 | 0.7823 | 0.7704 | 0.7823 | 0.7704 |
| 7 | 0.9991 | 0.9936 | 0.2801 | 0.2789 | 0.3866 | 0.3786 | 0.2801 | 0.2789 | 0.7823 | 0.7780 | 0.7823 | 0.7780 |
| 8 | 0.9997 | 0.9973 | 0.2803 | 0.2797 | 0.3888 | 0.3845 | 0.2803 | 0.2797 | 0.7819 | 0.7806 | 0.7819 | 0.7806 |
| 9 | 0.9999 | 0.9989 | 0.2804 | 0.2801 | 0.3897 | 0.3876 | 0.2804 | 0.2801 | 0.7815 | 0.7813 | 0.7815 | 0.7813 |
| 10 | 1.0000 | 0.9995 | 0.2805 | 0.2803 | 0.3902 | 0.3891 | 0.2805 | 0.2803 | 0.7812 | 0.7813 | 0.7812 | 0.7813 |

Supplementary Table 7a: Results from Copasi using the original SBML model of the gene regulatory network and the default set of initial parameter values.

| Generated by MATLAB | | | | | | | | | | | | |
|---|---|---|---|---|---|---|---|---|---|---|---|---|
| Time | pp1_mrna | p1 | pp2_mrna | p2 | pp3_mrna | p3 | pp4_mrna | p4 | pp5_mrna | p5 | pp6_mrna | p6 |
| 0 | 0 | 1 | 0 | 1 | 0 | 1 | 0 | 1 | 0 | 1 | 0 | 1 |
| 1 | 0.6321 | 0.6321 | 0.1580 | 0.4337 | 0.1622 | 0.4346 | 0.1580 | 0.4337 | 0.3950 | 0.5232 | 0.3950 | 0.5232 |
| 2 | 0.8647 | 0.7293 | 0.2271 | 0.2872 | 0.2519 | 0.2970 | 0.2271 | 0.2872 | 0.6204 | 0.5326 | 0.6204 | 0.5326 |
| 3 | 0.9502 | 0.8507 | 0.2605 | 0.2629 | 0.3145 | 0.2929 | 0.2605 | 0.2629 | 0.7265 | 0.6318 | 0.7265 | 0.6318 |
| 4 | 0.9817 | 0.9268 | 0.2739 | 0.2670 | 0.3523 | 0.3216 | 0.2739 | 0.2670 | 0.7672 | 0.7088 | 0.7672 | 0.7088 |
| 5 | 0.9933 | 0.9663 | 0.2784 | 0.2733 | 0.3722 | 0.3490 | 0.2784 | 0.2733 | 0.7796 | 0.7511 | 0.7796 | 0.7511 |
| 6 | 0.9975 | 0.9851 | 0.2797 | 0.2771 | 0.3820 | 0.3676 | 0.2797 | 0.2771 | 0.7823 | 0.7704 | 0.7823 | 0.7704 |
| 7 | 0.9991 | 0.9936 | 0.2801 | 0.2789 | 0.3866 | 0.3786 | 0.2801 | 0.2789 | 0.7823 | 0.7780 | 0.7823 | 0.7780 |
| 8 | 0.9997 | 0.9973 | 0.2803 | 0.2797 | 0.3888 | 0.3845 | 0.2803 | 0.2797 | 0.7819 | 0.7806 | 0.7819 | 0.7806 |
| 9 | 0.9999 | 0.9989 | 0.2804 | 0.2801 | 0.3897 | 0.3876 | 0.2804 | 0.2801 | 0.7815 | 0.7813 | 0.7815 | 0.7813 |
| 10 | 1.0000 | 0.9996 | 0.2805 | 0.2804 | 0.3902 | 0.3891 | 0.2805 | 0.2804 | 0.7812 | 0.7813 | 0.7812 | 0.7813 |

Supplementary Table 7b: Results from Copasi using the original SBML model of the gene regulatory network and the default set of initial parameter values.
